# Contributions to the knowledge of slugs and semi-slugs (Gastropoda, Pulmonata, Stylommatophora) from Algeria

**DOI:** 10.3897/zookeys.1270.156458

**Published:** 2026-02-27

**Authors:** Ramdane Ramdini, Fabio Liberto, Ghania Sadouk, Armando Gregorini, Mariastella Colomba

**Affiliations:** 1 Laboratoire de Production, sauvegarde des espèces menacées et des récoltes, Influence des variations climatiques, Mouloud Mammeri University, Tizi-Ouzou, Algeria University of Urbino Carlo Bo Urbino Italy; 2 Via del Giubileo Magno 93, 90015 Cefalù, Italy Mouloud Mammeri University Tizi-Ouzou Algeria; 3 Department of Biomolecular Sciences, University of Urbino Carlo Bo,Via Maggetti 22, 61029, Urbino, Italy Unaffiliated Cefalù Italy

**Keywords:** Check-list, distribution, North Africa, taxonomy

## Abstract

Seventeen species of slugs and semi-slugs from northern Algeria are described and discussed. The species are provided with synonyms, brief descriptions of external characters and genitalia, distribution, and ecology. Some taxonomic and biogeographical issues are discussed, and a key to the species is given. The paper is based on the analysis of material collected in North Algeria between 2018 and 2022 and on a critical review of the available literature data. *Ambigolimax parvipenis* is recorded for the first time from Algeria. *Limax brondelianus* Bourguignat, 1861 is regarded as a valid species of *Deroceras* as D. cf. brondelianum**comb. nov**. Two new synonyms are proposed: Amalia cabiliana Pollonera, 1891, **syn. nov**. is proposed as a junior synonym of *Milax gagates* (Draparnaud, 1801); *Ariunculus pallaryi* Collinge, 1904, **syn. nov**. is proposed as a junior synonym of *Letourneuxia nyctelia* Bourguignat, 1861.

## Introduction

Slugs and semi-slugs are not phylogenetic groups and do not constitute formal taxa. Slugs comprise various land snails in which the shell is reduced in size and concealed within the mantle. Semi-slugs include land snails whose shell is too small to contain the entire adult animal and remains visible at the back of the body, where it protects certain organs. External morphology and vestigial shell features often provide little useful information for classification, whereas examination of genital structures and molecular analyses can yield reliable data for accurate species classification.

Although interesting contributions have been published on northwestern African slugs in recent decades (Wiktor 1983a, b; Wiktor and Abbes 2008; Abbes et al. 2010; Martínez-Ortí and Borredà 2012, 2013; Borredà and Martínez-Ortí 2017; Hutchinson et al. 2022), the state of knowledge on these land molluscs is still unsatisfactory. Between the mid-19^th^ and early 20^th^ centuries, numerous slug taxa were described from Algeria, but most reports focused exclusively on the external appearance of the specimens or provided inadequate descriptions of the genitalia. Moreover, the reference material is limited because, in the 19^th^ century, slug specimens were rarely preserved in alcohol. Consequently, the taxonomic status of some Algerian slug nominal taxa remains uncertain at the species level and, in some cases, even at the genus level. When type series are unavailable, anatomical studies of topotypes and/or analysis of material from the entire distribution area are crucial to clarify the status of uncertain taxa. This paper reports and discusses a checklist of Algerian slugs and semi-slugs based on a critical review of available literature data and anatomical studies on recently sampled material.

### Study area

Algeria, with 2,381,741 km^2^, is the largest country in Africa. It can be divided into two distinct geographical regions, northern and southern. The northern region consists largely of the coastal Mediterranean plains and the Tell Atlas Mountains; the southern region makes up the majority of the country’s territory and consists almost entirely of the Sahara Desert. Northern Algeria has a typical Mediterranean climate, with warm, dry summers and mild, rainy winters. Total annual precipitation (TAP) increases along the coast from west to east, ranging from 400 to 670 mm. In the Kabylia and Edough regions, the TAP reaches ~ 1000 mm, and the continental Mediterranean climate dominates the Djurdjura Mountains, with heavy snowfall in winter. The coastal Mediterranean plains are the most fertile regions of the country, mainly cultivated with vineyards, olive groves, and citrus groves, and are inhabited by 90% of Algeria’s population. Forests cover only ~ 2% of Algeria and are mainly found in the less accessible regions of the Tell Mountains. These forests consist of holm oak, cork oak, juniper, and limited patches of cedar. Much of the Tell Atlas region was once covered by forest, but most of it has been deforested and replaced by scrubland or garigue. Northern Algeria has an interesting slug and semi-slug fauna, with some endemic and little-known species, which is the subject of this study.

The region immediately south of the coastal mountains has hot summers and cold winters, annual rainfall varies from 100 to 400 mm, and the vegetation is mainly steppe. In the desert, vegetation is sparse and highly dispersed, with species specialised for the extreme conditions typical of the desert. These regions are inhospitable to slugs and semi-slugs and were therefore excluded from this study, although some scattered reports are known from the Sahara oases (Abbes et al. 2010).

### Historical background

The first records of slugs and semi-slugs from Algeria were published by Forbes (1838: 251), Terver (1839: 9), Rossmässler (1841: 249), Morelet (1853: 280), and Debeaux (1857: 320); nevertheless, their species lists lack adequate descriptions of the taxa, making the data reported hardly verifiable. The first semi-slug species described from Algeria was Drusia (Escutiella) deshayesii (Moquin-Tandon, 1848). Its external and internal morphology was described in detail by Fischer (1856) and, a few years later, by Bourguignat (1862a). Between 1862 and 1866, Bourguignat published a series of papers dealing with all genera of slugs and semi-slugs living in Algeria and described thirteen new taxa. In the first version of his paper “Des limaces algériennes”, Bourguignat (1861) summarised previously published data and discussed a list of six species; in particular, he reported *Milax gagates* (Draparnaud, 1801) and five new species, including *Limax deshayesi*, today considered synonymous with *Limacus flavus* (Linnaeus, 1758) (Taylor 1902; Hesse 1926); *L. brondelianus* a species of *Deroceras* Rafinesque, 1820 requiring revision, *L. eremiophilus* Bourguignat, 1861 a species of *Milax* J. E. Gray, 1855 requiring revision, *L. raymondianus* (Bourguignat, 1861) a species of uncertain genus, and *L. nyctelius* accepted for many decades as *Lehmannia* Heynemann, 1863 or *Ambigolimax* Pollonera, 1887 and recently assigned to the genus *Letourneuxia* Bourguignat, 1866 as *Letourneuxia nyctelia* (Hutchinson et al. 2022).

Then, Bourguignat (1862b) published in the book “Les Spiciléges Malacologiques” the second version of “Des limaces algériennes”, adding two plates and describing two additional new species, *Limax subsaxanus*, considered to belong to the genus *Deroceras*, and *L. scaptobius*, a species considered to be part of the genus *Milax* (later illustrated in Bourguignat 1864: pl. 3, figs 13–16). Both names require modern revisions (Liberto et al. 2017).

In the same year, Bourguignat (1862c) published his monograph on the genus *Testacella* Lamarck, 1801, describing two new species from Algeria: *T. fischeriana*, currently accepted as an Algerian-Tunisian species, and *T. brondeli*, considered as its synonym (see Giusti et al. 1995).

Later, Bourguignat (1866), in the seventh issue of his work “Mollusques nouveaux, litigieux ou peu connus”, described the new genus and species *Letourneuxia numidica* and the new species *Daudebardia letourneuxi*. In the eleventh issue, published in 1870 in the journal “Revue et Magasin de Zoologie”, Bourguignat (1870) described two additional *Daudebardia* W. Hartmann, 1821 species: *D. nubigena* and *D. atlantica*.

To these three Algerian *Daudebardia* taxa, Letourneux (1870) added two new species, *D. platystoma* and *D. charopia*, from Kabylia (north Algeria). All five *Daudebardia* species were described based on their shell features alone.

In the last decade of the 1800s, four Milacidae taxa were described based on the external appearance of the specimens or with an inadequate and/or partial description of the genitalia: Amalia mediterranea Cockerell, 1891 (type locality: Algeria and Sicily); A. insularis var. algerica Pollonera, 1891 (t.l. Algiers); *A. cabiliana* Pollonera, 1891 (t.l. El-Hammam, Kabylia, Algeria); and *A. maculata* Collinge, 1895 (t.l. Algiers) . The latter name was preoccupied by *A. maculata* Koch & Heynemann, 1874; therefore, Hesse (1926) published the replacement name *A. collingei*.

Many authors have published distribution data for Algerian slugs, although the identifications which are not supported by anatomical descriptions and figures are of questionable value: Bourguignat (1864), Lallemant (1869, 1881), Letourneux (1870, 1872), Kobelt (1877), Brognart (1882), Pechaud (1884), Tryon (1885), Heynemann (1885), Pollonera (1890a, 1891), Taylor (1902–1907), Llabador (1936). Morphological data useful for a reliable classification and distribution of slug and semi-slug species from Algeria were published between the end of the 19^th^ and 20^th^ centuries. Simroth (1885), Connolly (1939), and Quick (1960) provided the first anatomical data on *Ambigolimax* species. Riedel (1978), in his re-examination of the subfamily Daudebardiinae, provided the first anatomical data on *D. rufa atlantica*. Wiktor (1983a) described the genitalia of *Letourneuxia* and introduced the new species *Deroceras riedelianum*; however, he did not consider the two older Algerian taxa *D. brondelianum* (Bourgignat, 1861) and *D. subsaxanum* (Bourgignat, 1862). Finally, Giusti et al. (1995) revised the genus *Testacella* and described the new Algerian-Maltese species *T. riedeli*.

Martínez-Ortí and Borredà (2012) revised the family Parmacellidae and assigned the species from western Algeria and eastern Morocco to the genus *Drusia* J. E. Gray, 1855 and to the new subgenus *Escutiella* Martínez-Ortí & Borredà, 2012.

Borredà and Martínez-Ortí (2017) published a list of slugs and semi-slugs from Maghreb with comments on 17 species, ten of which were listed from Algeria: Drusia (Escutiella) deshayesii; *Milax gagates*; *M. nigricans* (R. A. Philippi, 1836); *Lehmannia nyctelia*; *L. valentiana* (A. Férussac, 1821), *Limacus flavus*; *Deroceras ponsonbyi* (P. Hesse, 1884); *Letourneuxia numidica*; *Daudebardia rufa* (Draparnaud, 1805); and *D. brevipes* (Draparnaud, 1805).

Ramdini et al. (2021) recorded five slug species from the Kabylia region (northern Algeria): *Milax gagates*, *M. nigricans*, *Ambigolimax nyctelius*, *Testacella riedeli* and *Deroceras cf. riedelianum*. They illustrated the genitalia of a *Deroceras cf. riedelianum* which differ from the type series described by Wiktor (1983a) by having a less elongated penis and lacking both the process and the appendix-like distention in the proximal part of the penis.

Finally, Hutchinson et al. (2022), in their taxonomic revision of the genera *Ambigolimax* and *Letourneuxia*, ascertained the presence of *A. melitensis* from Algeria (Constantina). Based on these three recent studies, the number of previously confirmed species of slugs and semi-slugs from Algeria is eleven.

In this paper, we report and discuss the results of the morphological analysis of specimens collected in northern Algeria between 2018 and 2022. We reviewed the literature data, which allowed us to clarify some taxonomic issues concerning the Algerian slugs and semi-slugs, redefine their distribution area, and update the checklist.

## Materials and methods

### Taxon sampling

All specimens were collected on the ground and under logs, stones, and within the leaf-litter layer of forests. The size, external morphology, and genital morphology were investigated. To study and illustrate genitalia, specimens were fixed in 75% ethanol. The reproductive apparatus was extracted using a scalpel, scissors, and needles and studied under a stereomicroscope (Optika ST-156). Photographs were taken with a digital camera (OPTIKAHDMI Easy camera). The maximum length and width of the molluscs, together with the shell and some parts of the genitalia, were measured in millimetres using a digital gauge or a millimetre scale. In the anatomical description, the proximal part is the part closest to the gonad, and the distal part is the part closest to the gonopore. The proximal female genitalia, sometimes shown in the plates, are not described because they are uninformative.

**Figure 1.**
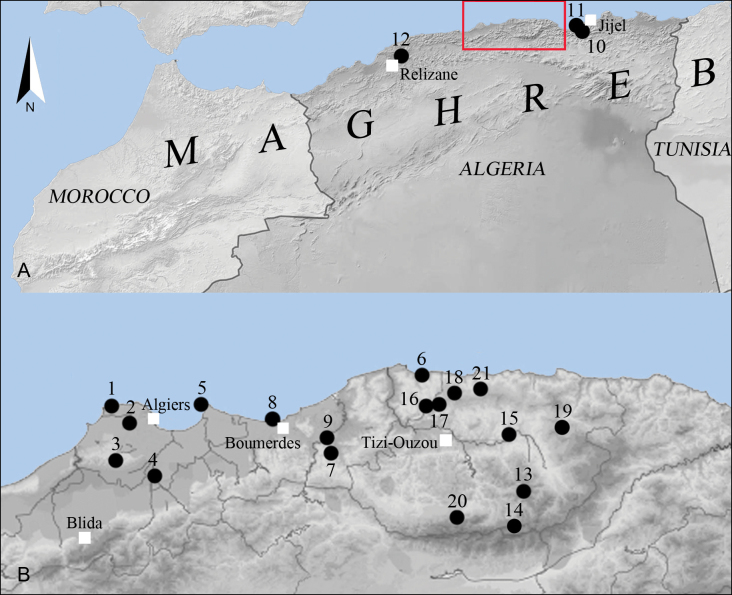
Map of sampled localities. **A**. Maghreb, in red the Kabylia region (northern Algeria), black dots = sampling stations, white squares = major cities; **B**. Detailed view of Kabylia Region.

Taxonomic references are based on MolluscaBase (2025) and other cited papers. Bibliographic references regarding Bourguignat’s publications are based on Bank et al. (2019). The systematic catalogue was organised using the following headings: taxon name, bibliographic references, material examined, diagnosis, distribution, ecology, and remarks. The collection localities were listed according to the following scheme: collecting station, geographic coordinates, altitude, and dates of collection. Voucher specimens used for this study are stored in the Ramdane Ramdini Collection (**RRC**), Laboratoire de production, sauvegarde des espèces menacées et des récoltes, influence des variations climatiques, Mouloud Mammeri University, Tizi-Ouzou, Algeria. Specimens were collected in Algeria from the following localities, listed in alphabetical order (Fig. 1A, B):

1. Algiers, Ain Benian; 36°48.267'N, 2°55.867'E; 25 m a.s.l.; December 2018.
2. Algiers, Dely Ibrahim; 36°45.233'N, 2°59.117'E; 260 m a.s.l.; December 2018.
3. Algiers, Douera; 36°39.750'N, 2°57.167'E; 180 m a.s.l.; January 2020.
4. Algiers, Sidi Moussa; 36°36.917'N, 3°5.317'E; 41 m a.s.l.; November 2020.
5. Algiers, Tamentfoust; 36°48.150'N, 3°14.350'E; 20 m a.s.l.; February 2020.
6. Boumerdès, Afir; 36°54.033'N, 3°58.350'E; 10 m a.s.l.; December 2019.
7. Boumerdès, Chabet El Ameur; 36°40.700'N, 3°40.117'E; 160–140 m a.s.l.; December 2019.
8. Boumerdès, Corso; 36°45.983'N, 3°26.817'E; 10 m a.s.l.; December 2019.
9. Boumerdès, Issers; 36°43.817'N, 3°39.733'E; 25 m a.s.l.; January 2020.
10. Jijel, National Park of Taza, Garouche forest; 36°40.033'N, 5°38.367'E; 700 m a.s.l.; January 2021.
11. Jijel, National Park of Taza, Tabola; 36°42.567'N, 5°33.000'E; 54 m a.s.l.; January 2021.
12. Relizane, El Hamadna; 35°53.633'N, 0°44.033'E; 85 m a.s.l.; 1 March 2022.
13. Tizi-Ouzou, Ain El Hammam; 36°34.283'N, 4°18.583'E; 1075 m a.s.l.; October 2019.
14. Tizi-Ouzou, Akvil, Ait Ouavan Forest; 36°29.017'N, 4°17.000'E; 855 m a.s.l.; 23 February 2022.
15. Tizi-Ouzou, El Kahra; 36°43.583'N, 4°15.867'E; 110 m a.s.l.; December 2020.
16. Tizi-Ouzou, Makouda, Tazarourt; 36°48.083'N, 4°1.367'E; 340 m a.s.l.; November 2020.
17. Tizi-Ouzou, Makouda, Tigoulmamine; 36°48.167'N, 4°1.867'E; 350 m a.s.l.; January 2021.
18. Tizi-Ouzou, Mizrana, Mizrana Forest; 36°50.050'N, 4°4.533'E; 730 m a.s.l.; January 2020.
19. Tizi-Ouzou, Yakouren, Yakouren Forest; 36°44.750'N, 4°26.100'E; 720 m a.s.l.; December 2020.
20. Tizi-Ouzou, Ouadhias; 36°30.017'N, 4°4.533'E; 750 m a.s.l.; February 2019.
21. Tizi-Ouzou, Tigzirt, Tifra; 36°50.667'N, 4°10.000'E; 630 m a.s.l.; October 2020.

## Results

**Class Gastropoda Cuvier, 1795**

**Infraclass Pulmonata Cuvier in Blainville, 1814**

**Order Stylommatophora A. Schmidt, 1855**

**Suborder Helicinae Rafinesque, 1815**

**Infraorder Limacoidei P. Fischer, 1856**

**Superfamily Parmacelloidea P. Fischer, 1856 (1855)**

**Family Milacidae Ellis, 1926**

### Genus Milax

Taxon classificationAnimaliaStylommatophoraMilacidae

J. E. Gray, 1855

C6483C02-81DA-50C8-946C-14467005DF51

#### Diagnosis.

Body in the Algerian species ≤ 50–100 mm, width 7–15 mm; mantle length 13–30 mm; keel very prominent, visible along the whole length of the back; mantle notched posteriorly near the keel and with a distinct horseshoe-shaped groove; pneumostome almost postmedial; shell nail-like, slightly convex dorsally, more or less calcified ventrally, the nucleus is placed posteriorly on the major axis, upper surface with concentric irregular growth lines, in ventral view a thick straight wall delimits the posterior margin of the shell; genitalia with a single, large accessory gland, opening laterally to atrium; large atrium with stimulator inside, duct of the bursa copulatrix opens on the vagina, sometimes on the atrium.

### Milax ater

Taxon classificationAnimaliaStylommatophoraMilacidae

(Collinge, 1895)

2F6FF9EF-02F5-57FB-AEC9-1D96BC6B2BCE

Figs 2, 3, 53

Amalia ater Collinge, 1895: 336, pl. 23, figs 1–5 – Algeria – (Anat.).
Milax gagates – Wiktor 1983a: 160 (partim).
Milax nigricans – Wiktor 1983a: 161 (partim).
Milax ater – Wiktor 1987: 193–196, figs 42–50, map. 2 – Algeria, Constantine, Gorges du Rhumel, and Tizi-Ougoulmime, Djurdjura Mts. – (Anat.).

#### Material examined.

• 2 exx; Tizi-Ouzou, Akvil, Ait Ouavan Forest; 36°29.017'N, 4°17.000'E; 855 m a.s.l.; 23 February 2022; R. Ramdini leg.

**Figures 2, 3.**
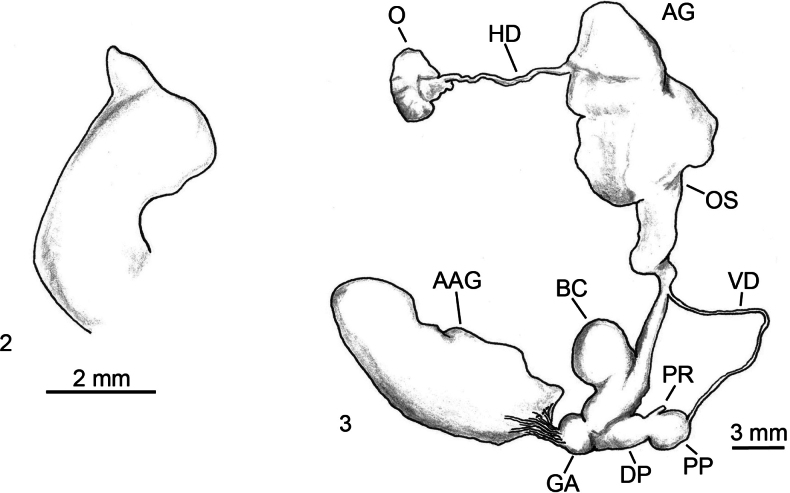
*Milax ater*, Algeria, Tizi Ouzou, Akvil, Ait Ouavan Forest. **2**. Stimulator; **3**. Genitalia. Abbreviations: AAA atrial accessory appendices, AAG atrial accessory gland, AG albumen gland, BC bursa copulatrix, DBC duct of the bursa copulatrix, DP distal penis, E epiphallus, GA genital atrium, HD hermaphrodite duct, LI ligule, ML mantle lobe, O ovotestis, OS ovispermiduct, P penis, PA penial appendix, PF penial flap, PP proximal penis, PPC proximal penial crest, PR penial retractor muscle, PRG perivaginal gland, PRO prostate, ST stimulator, V vagina, VG vaginal gland, VD vas deferens.

#### Diagnosis.

Medium-sized slug, body length 35–50 mm; mantle length 10–20 mm; body uniformly black, in some specimens head and sides downwards lighter, sole dirty creamy; oval-conical smooth stimulator, on its tip there are small flat processes or a sort of fan (Fig. 2). Penis roughly equal in length to epiphallus, a shallow constriction separates the penis from the epiphallus (Fig. 3); inside the epiphallus dense small papillae, penis papilla of simple structure; inside the penis complex folds of two types; spermatophore unknown.

#### Distribution.

Species endemic to Algeria: Constantina (Gorges du Rhumel); Tizi-Ougoulmime (Mountain of Djurdjura).

#### Ecology.

Collected on limestone and in humid places (Wiktor 1987), under stones and decomposing organic matter.

#### Remarks.

The slug is externally indistinguishable from the other two *Milax* species occurring in Algeria, *M. nigricans* and *M. gagates*. It can be reliably identified by examining the genital organs, particularly the characteristics of the stimulator. The two specimens examined showed a short, almost oval, smooth stimulator, one with a hook near the apex and the other with a blunt apex. In *M. gagates*, the stimulator is narrower, tongue-shaped, with a few irregularly scattered small, spiny papillae towards the apex, whereas in *M. nigricans* it is conical, with papillae randomly distributed on the inner side, most of them located at the stimulator base. *Milax ater* is endemic to Algeria and is known from only a few localities; it is a species deserving attention and protection.

### Milax gagates

Taxon classificationAnimaliaStylommatophoraMilacidae

(Draparnaud, 1801)

2E643846-1073-551F-989B-30E849530557

Figs 4–6, 54

Amalia cabiliana – Pollonera 1891 : 4 – El-Hammam nella Cabilia, Algeria. syn. nov.
Amalia gagates – Seurat 1930 : 260 – Algeria.
Milax gagates – Wiktor 1983a: 161 – Algeria: Ait Haouari, Djurdjura Mts., south of Mechtras; Tizi-Ougoulimime Djudjura Mts. 1770 m, west of Tikjda; Kabylia, mountains midway between Mirabeau and Dra-el-Mizan; Morocco: Kazba-Tadia – (Anat.). Wiktor 1987: 202–206, figs 67–72, map. 3 – Algeria, Morocco – (Anat.). Wiktor 1996: 20, fig. 14 – NW Africa. Abbes et al. 2010: 220–223 – Tunisia: Tinja; Djebel Ammar; Bni Mtir; Zaghouan; Ichkeul National Park; Zriba; Aïn Errahma; Aïn Draham; Bir Bouregba. Borredà and Martínez-Ortí 2017: 4, fig. 4 – Morocco: Aoufous, Tafilalelt; Tassout, Oued Lakhdar; Beni Melal; Tighssaline; Tiouririne, oued after Sidi Addi; Azrou; Larache; Spain: Melilla, Legion Barracks; Algeria: Road Tlemcen, Sebou, before to the junction to Beni Snous; Bejaïa, Cap Carbon, Pic des Signes; Tlemcen, Zariffet Forest; Oran, Ain el Turk; Algiers, Aïn Taya.

#### Material examined.

• 2 exx; Tizi-Ouzou, Ain El Hammam; 36°34.283'N, 4°18.583'E; 1075 m a.s.l.; October 2019; R. Ramdini leg. • 15 exx; Tizi-Ouzou, Makouda, Tazarourt; 36°48.083'N, 4°1.367'E; 340 m a.s.l.; November 2020; R. Ramdini leg. • 18 exx; Tizi-Ouzou, El Kahra; 36°43.583'N, 4°15.867'E; 110 m a.s.l.; December 2020; R. Ramdini leg. • 12 exx; Jijel, National Park of Taza, Garouche forest; 36°40.033'N, 5°38.367'E; 700 m a.s.l.; January 2021; R. Ramdini leg.

**Figures 4–6.**
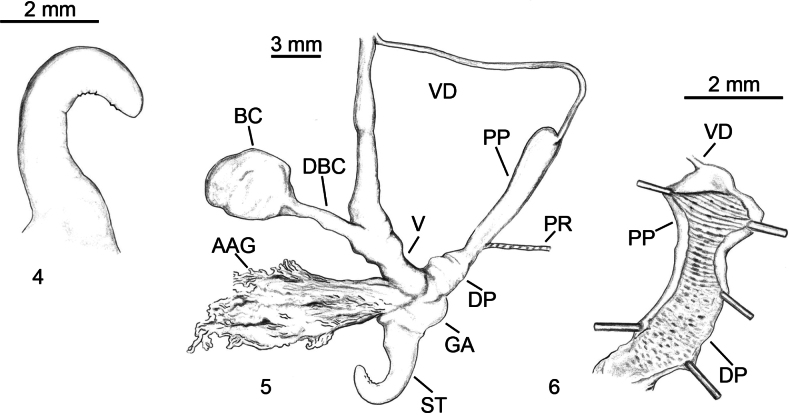
*Milax gagates*, Algeria, Tizi-Ouzou, Ain El Hammam. **4**. Stimulator; **5**. Genitalia; **6**. Internal view of penis.

#### Diagnosis.

Body uniformly blackish or dark grey; sole with blackish sides and lighter middle; stimulator narrow, tongue-shaped, gradually narrowing towards its end, smooth with few irregularly scattered, small, spiny papillae at the apex (Fig. 4); epiphallus claviform and double or more penis length (Fig. 5); internal walls of the epiphallus show a reticulated structure, with annular grooves in the proximal part (Fig. 6); spermatophore elongate, posterior end somewhat broader, spines branching dichotomously yet quite irregularly, covering one side of the spermatophore, the other side is smooth.

#### Distribution.

Southwestern Europe and northwestern Africa. Introduced to central and northern Europe, Tasmania, New Zealand, Polynesia, America, South Africa, Japan, Bermuda, and numerous islands on the Pacific and Atlantic Oceans (Herbert 2010 and cited literature).

#### Ecology.

This species was recorded in a variety of habitats, in grasslands under wet rocks, in forests under tree trunks and in shady, damp places; it was found at 1000 m altitude in Ain El-Hammam (Michelet, Tizi-Ouzou).

#### Remarks.

*Milax gagates* is a common species in Morocco and northwestern Algeria, less common in northeastern Algeria and Tunisia (Abbes et al. 2010; this study). Pollonera (1891) described the new species Amalia cabiliana (type locality El-Hammam, Kabylia, Algeria) with genitalia indistinguishable from those of *M. gagates* and differing only in external features (smaller body dimensions, paler groves on the back, and a band around the mantle groove less regular and less visible). Amalia cabiliana was subsequently reported by Pollonera (1916) from Dar M’tougui, Amismiz, Grand Atlas, Morocco. Specimens from the type locality examined by us turned out to be indistinguishable from *M. gagates*. We propose *A. cabiliana* Pollonera, 1891 as a junior synonym of *M. gagates* (Draparnaud, 1801).

### Milax nigricans

Taxon classificationAnimaliaStylommatophoraMilacidae

(R. A. Philippi, 1836)

23AB1915-3156-5F97-AA68-12CD3A11C80C

Figs 7–16, 55

Milax nigricans – Wiktor 1983a: 161 – Algeria: Hamma, Jardin d’Essai; Lakhdaria (= Gorges de Palestro), rocky river gape of Isser; Kabylia, Skikda (= Philippeville); Annaba (= Bone). Roman ruins of Hippona; Djebel Edough in the vicinity of Annaba (= Bone), between Seraidi (= Bugeaud) and Sainte-Croix-d’Edough. Wiktor 1987: 159, 161, 179, 193, 195, 205–207, 211–214, figs 78–84, map. 4 – Tunisia, Algeria, Morocco, Egypt – (Anat.). Wiktor 1996: 20–21, figs 15–19 – Tunisia, Algeria, Morocco, Egypt.

#### Material examined.

• 24 exx; Tizi-Ouzou, Yakouren, Yakouren Forest; 36°44.750'N, 4°26.100'E; 720 m a.s.l.; November 2018; R. Ramdini leg. • 15 exx; Tizi-Ouzou, Makouda, Tazarourt; 36°48.083'N, 4°1.367'E; 340 m a.s.l.; December 2018; R. Ramdini leg. • 9 exx; Algiers, Ain Benian; 36°48.267'N, 2°55.867'E; 25 m a.s.l.; December 2018; R. Ramdini leg. • 9 exx; Algiers, Dely Ibrahim; 36°45.233'N, 2°59.117'E; 260 m a.s.l.; December 2018; R. Ramdini leg. • 8 exx; Tizi-Ouzou, Ouadhias; 36°30.017'N, 4°4.533'E; 750 m a.s.l.; February 2019; R. Ramdini leg. • 6 exx; Boumerdès, Afir; 36°54.033'N, 3°58.350'E; 10 m a.s.l.; December 2019; R. Ramdini leg. • 7 exx; Boumerdès, Corse; 36°45.983'N, 3°26.817'E; 10 m a.s.l.; December 2019; R. Ramdini leg. • 11 exx; Boumerdès, Chabet El Ameur; 36°40.700'N, 3°40.117'E; 160/140 m a.s.l.; December 2019; R. Ramdini leg. • 10 exx; Tizi-Ouzou, Mizrana, Mizrana Forest; 36°50.050'N, 4°4.533'E; 730 m a.s.l.; January 2020; R. Ramdini leg. • 13 exx; Boumerdès, Issers; 36°43.817'N, 3°39.733'E; 25 m a.s.l.; January 2020; R. Ramdini leg. • 17 exx; Algiers, Douera; 36°39.750'N, 2°57.167'E; 180 m a.s.l.; January 2020; R. Ramdini leg. • 6 exx; Algiers, Tamentfoust; 36°48.150'N, 3°14.350'E; 20 m a.s.l.; February 2020; R. Ramdini leg. • 15 exx; Tizi-Ouzou, Tigzirt, Tifra; 36°50.667'N, 4°10.000'E; 630 m a.s.l.; October 2020; R. Ramdini leg. • 9 exx; Algiers, Sidi Moussa; 36°36.917'N, 3°5.317'E; 41 m a.s.l.; November 2020; R. Ramdini leg. • 19 exx; Tizi-Ouzou, Makouda, Tigoulmamine; 36°48.167'N, 4°1.867'E; 350 m a.s.l.; January 2021; R. Ramdini leg.

**Figures 7–16.**
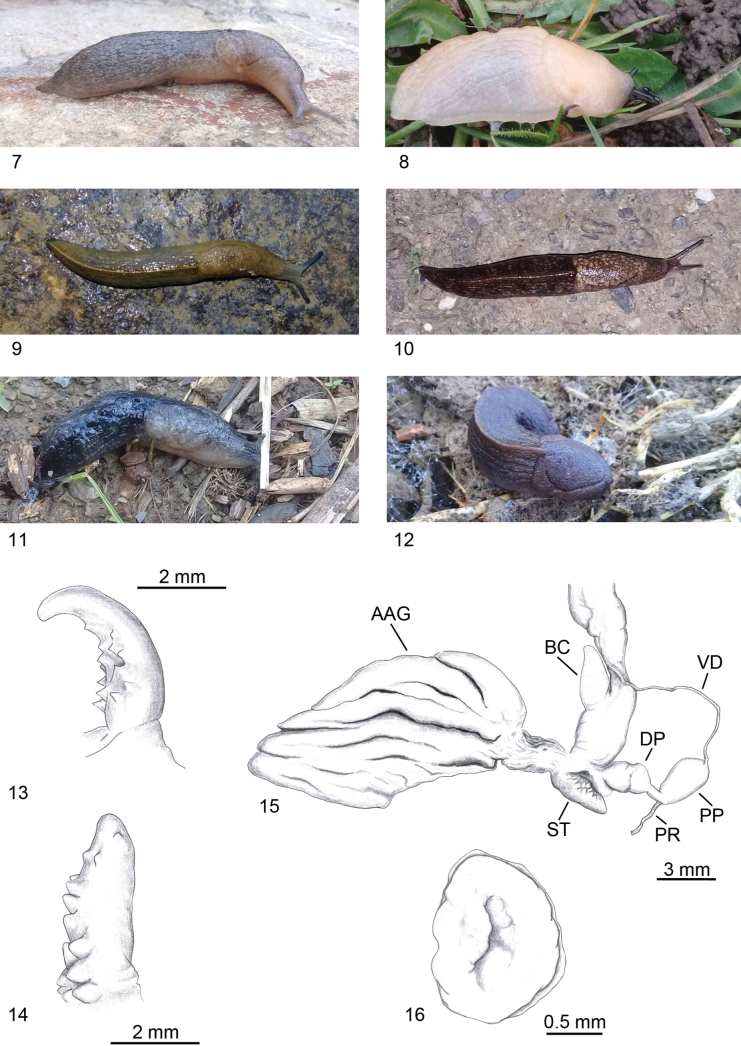
*Milax nigricans*. **7–12**. Colour variability in living specimens. **7**. Tizi-Ouzou, Makouda, Tazarourt; **8**. Algiers, Douera; **9**. Tizi-Ouzou, Makouda, Tigoulmamine; **10**. Tizi-Ouzou, Makouda, Tigoulmamine; **11**. Tizi-Ouzou, Tigzirt, Tifra; **12**. Tizi-Ouzou, Makouda, Tazarourt. **13–16**. Genitalia; **13**. Stimulator, Algeria, Tigoulmine, Makouda; **14**. Stimulator, Algeria, Boumerdes, Issers; **15**. Genitalia, Algeria, Tigoulmine, Makouda; **16**. Penial papilla, Algeria, Tizi-Ouzou, El Kahra.

#### Diagnosis.

While European populations show a blackish or pale greyish body and cream sole (Wiktor 1987; Turóci et al. 2023), the Algerian *M. nigricans* populations show remarkable variability in the external colouring: brown with paler spots, or uniform pale cream with dark head and tentacles, or grey with whitish spots, or uniform white (Figs 7–12). Genitalia characterised by stimulator conical with acuminate to rounded papillae randomly distributed on the inner side, most of them situated at the stimulator base and absent at its tip (Figs 13, 14); penis oval, epiphallus claviform, slightly longer than the penis (Fig. 15); short penial papilla of annular shape (Fig. 16); spermatophore elongate conical, covered on all sides and along its whole length by multiply branched spines, the largest and most dense spines cover the anterior ending.

#### Distribution.

This species, probably native to the Mediterranean area, currently has a European-Mediterranean distribution that extends to the Canary Islands (Wiktor 1987).

#### Ecology.

*Milax nigricans* is synanthropic; it colonises a wide range of habitats, gardens, greenhouses, parks, and cultivated land, as well as oak forests and olive groves. It takes refuge mainly under stones, organic debris, or any substrate capable of creating wet micro-habitats (plastic, polystyrene, paper).

#### Remarks.

Many taxa have been established for the genus *Milax* from Algeria. However, they were described based on the external morphology and colouring of the slugs, with little data or without any data on the diagnostic characters of the reproductive system and spermatophore. Our morphological examinations revealed a relevant colour variability in *M. nigricans* from Algeria, which is unknown in other regions. The other two Algerian *Milax* species, *M. ater* and *M. gagates*, have scarcely variable blackish or greyish colouring. This leads us to suspect that several older nominal taxa (based on external colouring) may be synonymous with *M. nigricans*. However, more intensive sampling is needed, and we prefer to keep them as taxa inquirenda. Taxa inquirenda in the genus *Milax* described from Algeria are gagates var. *mediterranea* Cockerell, 1891 (t.l. east Algeria and Sicily); eremiophilus Bourguignat, 1861 (t.l. surroundings of Cherchell); scaptobius Bourguignat, 1862 (t.l. surroundings of Bougie, surroundings of Constantine); insularis var. *algerica* Pollonera, 1891 (t.l. Algiers); collingei Hesse, 1926 (t.l. Algiers).

##### Family Parmacellidae P. Fischer, 1856 (1855)

**Genus *Drusia* J. E. Gray, 1855**

### Subgenus Escutiella

Taxon classificationAnimaliaStylommatophoraParmacellidae

Martínez-Ortí & Borredà, 2012

480D1D76-661A-5378-AB62-57102440FD9F

#### Diagnosis.

Body of medium size (length ≤ 60 mm, width ≤ 16 mm); grainy skin; short and keeled back; very wide and long mantle (half body length), crossed by a large semicircular groove, which on the right side runs forward passing through the pneumostome; the latter is in posterior position; internal shell situated under the posterior part of the mantle consisting of a short spiral part attached to a flat plate; genitalia characterised by penis with extrusion, epiphallus internally reticulated, two accessory atrial appendices slightly different in size.

### Drusia (Escutiella) deshayesii

Taxon classificationAnimaliaStylommatophoraParmacellidae

(Moquin-Tandon, 1848)

895AA0B0-0DA1-558B-A641-5F1297B05836

Figs 56–58

Parmacella deshayesi – Moquin-Tandon 1848: 261, pl. 1, fig. 5. Moquin-Tandon 1850: 49, 54, 55, 56 – province d’Oran. Fischer 1856: 380–398, pl. 5 – Oran. Bourguignat 1861: 47–54, pls. 4, 5 – Environs d’Oran (Algerie). Blanchard 1891: 215 – Algeria: Orléansville. Seurat 1930: 261 – Algérie. Llabador 1936: 201, 203 – Algeria: Massif des M’sirdas au Sud-Ouest de Nemours.
Parmacella dorsalis – Pechaud 1884: 8 – Au village Lamoricière, entre Tlemcen et Sidi-bel-Abbès; Tanger – Not Moquin-Tandon 1848.
Parmacella valenciennesi – Hesse 1885: 9–12 (sic!) – Morocco: Tanger – (Anat.) – (Not Webb & Van Beneden, 1836).
Parmacella (Parmacella) deshayesi – Wiktor 1983b: 83–85, figs 1–9 – Morocco: Sidi Slimane, oued Elhadar, 3 km S from Taza Haut; Beni Hozmar Mts, ca. 5 km S. of Tetuan.
Parmacella (Escutiella) deshayesii – Martínez-Ortí and Borredà 2012: 1–18, figs 1a-f, 2a-d, 3a-i, 5a-e, 6 (map) – Oran, Ain Franin, Oran-Kristel road, near the cliff (UTM=YE2765); Tlemcen (UTM=XD7372).
Drusia (Escutiella) deshayesii – Borredà and Martínez-Ortí 2017: 4, fig. 3 – Ain Franin, road Oran- Kristel, near the diff; Tlemcen.

#### Material examined.

• 5 exx; Relizane, El Hamadna; 35°53.633'N, 0°44.033'E; 85 m a.s.l.; 1 March 2022; A. Bekkouche leg.

#### Diagnosis.

Body unicolour reddish-brown both in juveniles and adults, with no bands or spots; shell with smooth protoconch and wide, oval spatula; genitalia characterised by a long vas deferens; gradually widening epiphallus; cylindrical penis with a swelling in the middle; short atrium, atrial accessory appendices only slightly unequal and with a big fold inside; very large perivaginal gland; duct of the bursa copulatrix widened at the base; large oval bursa copulatrix.

#### Distribution.

Northwest Algeria and northeast Morocco.

#### Ecology.

Parmacellids are herbivorous.

#### Remarks.

*Drusia deshayesii* is a well-known species (Wiktor 1983b; Martínez-Ortí and Borredà 2012).

##### Superfamily Limacoidea Batsch, 1789

**Family Limacidae Lamarck, 1801**

**Subfamily Limacinae Lamarck, 1801**

### Genus Limacus

Taxon classificationAnimaliaStylommatophoraParmacellidae

Lehmann, 1864

FFF1CDA7-7DB1-5F76-8015-E81841B3C2F0

#### Diagnosis.

Body length ≤ 120 mm; keel short and ill-defined; mantle covers < 1/3 of body length; pneumostome postmedial; body yellowish or greenish-brown, with yellowish spots; shell thin, oblong-oval, dorsal surface weakly convex with distinct growth lines, ventral surface concave; protoconch not protruding, positioned asymmetrically on the left side; organic layer surrounding the shell, partially decalcified shells not uncommon; prostate longer than the oviduct and not fused to it at the anterior end.

### Limacus flavus

Taxon classificationAnimaliaStylommatophoraParmacellidae

(Linnaeus, 1758)

2393B1B2-158F-509F-B7A3-FCC35150F63C

Figs 63–65

Limax deshayesi – Bourguignat 1861: 36–37, pl. 1, figs 1–2 – Environs de Cherchell et les jardins qui avoisinent Alger. Bourguignat 1864: 37, pl. 1, figs 3–4 – Environs de Cherchell; Alger. Lallemant 1869: 23 – Environs d’Alger. Lallemant 1881: 83 – Algérie: Alger. Brognart 1882: 1089 – Atlas au-dessus de Blida. Heynemann 1885: 258 – Algeria: Cherchell, Algiers, Oran bis Sagda.
Limax (Limax) flavus – Tryon 1885: 200, pl. 49, figs 70–72; pl. 50, fig. 76 – Algiers.
Limax (Plepticlimax) flavus – Pollonera 1891: 1–2 – Cherchell, Algeri, Bona.
Limax flavus – Taylor 1903: 88 – Morocco: Tangiers; Algeria: Cherchell, Algiers; Libya: Tripoli. Seurat 1930: 260 – Algeria.

#### Material examined.

No specimens in our samples.

#### Diagnosis.

Penis is cylindrical, without an appendix, measuring ~ 1/6 of body length, with an internal proximal penis wall with 3–6 longitudinal wavy flaps, followed in the distal penis by three larger folds, one of which is expanded, bursa copulatrix duct joined with the vagina (Abbes et al. 2010).

#### Distribution.

The original distribution area of *L. flavus* is not known precisely; probably in southeastern Europe. Nowadays, *L. flavus* has been introduced worldwide.

#### Ecology.

A species tolerant of human disturbance, which inhabits gardens and parks, recorded near human dwellings, especially basements and cellars (Borredà and Martínez-Ortí-2017).

#### Remarks.

*Limacus flavus* was recorded especially as a synanthropic species in Algeria.

### Genus Ambigolimax

Taxon classificationAnimaliaStylommatophoraParmacellidae

Pollonera, 1887

548966F1-E80E-59D6-B51A-49932A61067D

#### Diagnosis.

Body length ≤ 80 mm; mantle not more than 1/3 of body length; pneumostome postmedial; keel short, ill-defined; shell oval, nail-like, varying in degree of calcification, with lateral nucleus; penis short ≤ 2/3 of mantle, with or without an undivided appendix, inside the penis are one or two folds; bursa copulatrix connected with penis; prostatic gland fused to oviduct for the gland’s entire length.

### Ambigolimax melitensis

Taxon classificationAnimaliaStylommatophoraParmacellidae

(Lessona & Pollonera, 1882)

AD48EBB8-5B2F-5FCB-9731-F3EF0B66CEA2

Fig. 68

Ambigolimax melitensis – Hutchinson 2022: 21–22, fig. 2A-C – Algeria, Constantine.

#### Material examined.

No specimens in our samples.

#### Diagnosis.

Body slender, length ≤ 40 mm; mantle covers at most 1/3 body length, posteriorly subangular, with a superficial pattern of fingerprint-like, concentric rings; pneumostome at the posterior half of the mantle; keel limited to the posterior body end. Body colour ranges from pale grey to pink, often with two lateral longitudinal dark brown bands. Penis sac-like, C-shaped, with long, slender, tapering flagellum entering penis tip laterally, at a small distance from where the very short vas deferens ends; penial retractor muscle ending on the penis ~ 1/3 from the tip; penis with internal S- or C-shaped crest, starting from near where vas deferens enters the penis, running distally, then turning backwards to end about halfway along penis; bursa copulatrix duct arising from distal penis (Giusti et al. 1995).

#### Distribution.

Italy (peninsular Italy, Sicily, and Sardinia), France (Corsica), the Maltese Islands, northern Tunisia, and northern Algeria.

#### Ecology.

It inhabits damp microhabitats, both natural and anthropised (Giusti et al. 1995).

#### Remarks.

This species, previously regarded as *Lehmannia*, was recently allocated to the genus *Ambigolimax* by Hutchinson et al. (2022: 20). *Ambigolimax melitensis* is confirmed in Algeria based on specimens preserved in the Muséum d’histoire naturelle, Geneva and the State Museum of Natural History, Stuttgart (Hutchinson et al. 2022).

### Ambigolimax waterstoni

Taxon classificationAnimaliaStylommatophoraParmacellidae

Hutchinson, Reise & Schlitt, 2022

B741223F-2120-51D6-A9A8-81D48A646DE3

Figs 17–20, 69

Limax nyctelius – Connolly 1939: 176–177 – Algeria, Morocco – not Bourguignat 1861. Quick 1960: 200–201, fig. 17 B, C – Shebin El Kom, Egypt; Algeria – not Bourguignat 1861.
Limax (Limacus) nyctelius – Regteren Altena 1966: 190–192 – Algeria – not Bourguignat 1861
Lehmannia nyctelia – Wiktor 1983a: 161–162 – Algeria: Hamma. Jardin d’Essai; Lakhdaria (= Gorges de Palestro), rocky gape of the river Isser; Defile Sebaou near Tizi Ouzou Kabylia; Kabylia, mountains halfway between Mirabeau and Dra-el-Mizan – not Bourguignat 1861
Ambigolimax waterstoni – Hutchinson et al. 2022: 38–40, figs 5, 6, 12B, C.

#### Material examined.

• 2 exx; Boumerdès, Corso; 36°45.983'N, 3°26.817'E; 10 m a.s.l.; January 2019; R. Ramdini leg. • 2 exx; Tizi-Ouzou, Akvil, Ait Ouavan forest; 36°29.017'N, 4°17.000'E; 855 m a.s.l.; February 2022; R. Ramdini leg.

**Figures 17–20.**
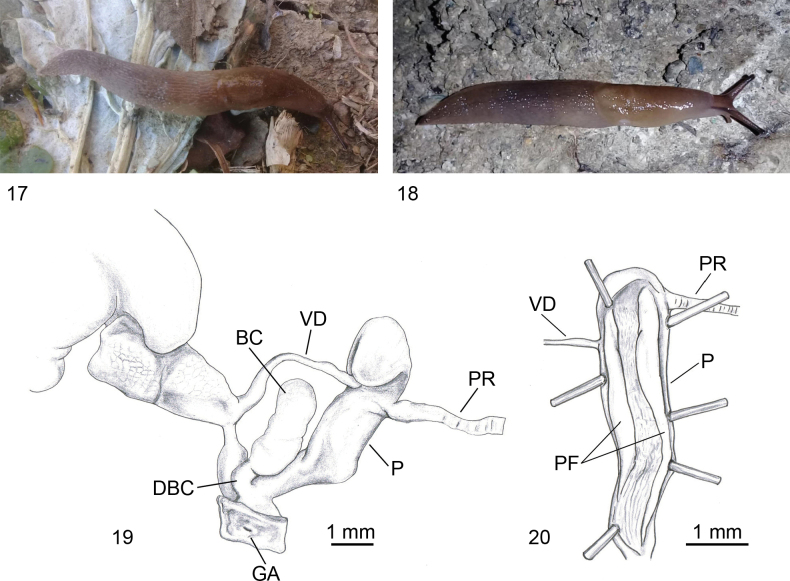
*Ambigolimax waterstoni*. **17**. Living specimen, Algeria, Tizi-Ouzou, Makouda, Tigoulmamine; **18**. Living specimen, Algeria, Tizi-Ouzou, Makouda, Tigoulmamine; **19**. Genitalia, Algeria, Boumerdès, Corso; **20**. Internal view of penis, same specimen as in Fig. 19.

#### Diagnosis.

Body slender, length ≤ 55 mm; mantle and keel like the previous species, brown to yellowish with two dark bands on the mantle and posterior body sides; occasionally, there is an additional, more or less clear band along the middle of the mantle (Figs 17, 18). Genitalia characterised by a long (4–6 mm) cylindrical penis, often swollen at the apex; the short vas deferens and the penial retractor muscle enters near penial apex beside one another; duct of the bursa copulatrix arising from the distal penis (Fig. 19); inside the penis two flaps originate from the penial apex and run parallel, decreasing in height towards the atrium (Fig. 20) (Connolly 1939; Quick 1960; Giusti 1976; Barker 1979; Wiktor 1983a; Hutchinson et al. 2022).

#### Distribution.

*Ambigolimax waterstoni* is presumed native to North Africa, but it is known as an alien species from Scottish greenhouses, Elba Island (Italy), the USA, South Africa, Australia, and New Zealand (Herbert 2010; Hutchinson et al. 2022).

#### Ecology.

Found in humid places, under stones, it can be synanthropic.

#### Remarks.

This species has been accepted for many decades under the name *Lehmannia nyctelia* or *Ambigolimax nyctelius*; however, Hutchinson et al. (2022) proposed the taxon *nyctelia* Bourguignat, 1861 as not available for *Lehmannia* or *Ambigolimax*. Indeed, the description and illustration of the pneumostome in the anterior part of the mantle (Bourguignat 1861) unambiguously refer nyctelius to the arionid genus *Letourneuxia*. Hutchinson et al. (2022) introduced the new name *A. waterstoni* for the North African native *Ambigolimax* species (previously named *A. nyctelia*) characterised by a long cylindrical penis, two parallel folds inside the penis, vas deferens, and penial retractor muscle entering near the penial apex.

### Ambigolimax valentianus

Taxon classificationAnimaliaStylommatophoraParmacellidae

(A. Férussac, 1821)

989801E6-9AA2-51E2-B7D8-F0811F76E770

Fig. 70

Limax valentianus – Letourneux 1870: 279 – Bone, Edough.
Lehmannia valentiana – Wiktor 1983a: 162 – Algeria: Djurdjura Mts., cedar forest Ait Ouboane, ca 1670 m. near Tizi N’Kouilal Pass; Djurdjura Mts., Tizi Ougoulmime Pass, ca 1700 m, west of Tikjda, Bouira; Djebel Edough near Annaba (= Bone); between Seraidi (= Bugeaud) and Sainte-Croix-d’Edough; Constantine, Gorges du Rhumel, at the bottom of defile. Borredà and Martínez-Ortí 2017: Morocco, Road Agadir, Essaouira, 29RMQ2470 08/1999; Algeria: Bejaïa, Cap Carbon, Pic des Signes, 32SJF5076, 03/2008; Algiers, Aïn Taya, 31SEA2372, 2009.

#### Material examined.

No specimens in our samples.

#### Diagnosis.

The body is very similar to other *Ambigolimax* species and is distinguishable with certainty only through examining the genitalia. Genitalia characterised by a penis claviform, posteriorly irregularly broadened, with a diagnostic short, blunt penial appendix, the latter arising laterally from the proximal penis, in the neighbourhood of vas deferens insertion; a V-shaped fold inside the penis, arising from the base of the appendix and running distally.

#### Distribution.

*Ambigolimax valentianus* is considered native to the Iberian Peninsula and northwest Africa; however, it has been spread by human activities to many other parts of the world (Herbert 2010 and cited literature; Sparacio et al. 2018).

#### Ecology.

Synanthropic species (South 1992), often found in gardens, nurseries, and greenhouses, specimens take refuge under woods and wet litter. In Algeria, it inhabits shady environments and cedar and oak forests (Wiktor 1983a).

#### Remarks.

Letourneux (1870: 279) was the first author to report *A. valentianus* from northeastern Algeria (Bone, Edough). Wiktor (1983a) confirmed it through anatomical examinations. Hutchinson et al. (2022) found a specimen confirmed as belonging to this species in the Bourguignat collection labelled “Foret de l’Edough, près Bône”. It is quite likely that Bourguignat obtained it from Letourneux. The specimen was labelled as nyctelius var. *major* (which Letourneux also listed). Abbes et al. (2010) recorded *L. marginata* from northern Tunisia. They likely misidentified specimens of *A. valentianus*, since *L. marginata* is widespread in central and western Europe and prefers woodland habitats, which differ from those in northern Tunisia (Borredà and Martínez-Ortí 2017).

### Ambigolimax parvipenis

Taxon classificationAnimaliaStylommatophoraParmacellidae

Hutchinson, Reise & Schlitt, 2022

674A20FF-E18A-5DD8-A14A-AF1FE2D84D2A

Figs 21, 22, 23, 24, 25, 26, 27, 28, 71

Lehmannia nyctelia – Borredà and Martínez-Ortí 2017: 4, fig. 5, Hutchinson et al. (2022: 31) – Spain: Chafarinas islands – Not Bourguignat 1861.

#### Material examined.

• 3 exx; Tizi-Ouzou, Mizrana, Mizrana Forest; 36°50.050'N, 4°4.533'E; 730 m a.s.l.; January 2020; R. Ramdini leg. • 10 exx; Algiers, Dely Ibrahim; 36°45.233'N, 2°59.117'E; 260 m a.s.l.; January 2020; R. Ramdini leg. • 6 exx; Tizi-Ouzou, Makouda, Tigoulmamine; 36°48.167'N, 4°1.867'E; 350 m a.s.l.; October 2020; R. Ramdini leg. • 14 exx; Tizi-Ouzou, Yakouren, Yakouren Forest; 36°44.750'N, 4°26.100'E; 720 m a.s.l.; November 2020; R. Ramdini leg.

**Figures 21–25.**
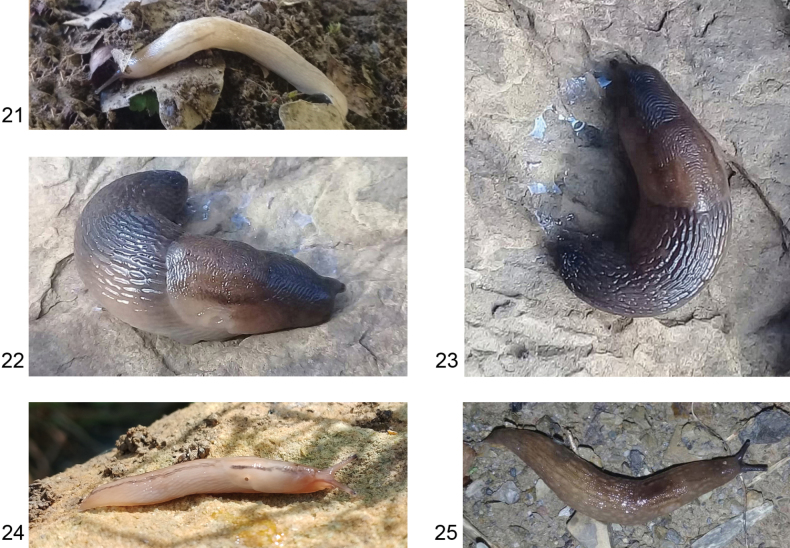
Colour variations of *Ambigolimax parvipenis*. **21**. Tizi-Ouzou, El Kahra, Tamda; **22, 23**. Tizi-Ouzou, Makouda, Tigoulmamine; **24**. Tizi-Ouzou, Makouda, Tazarourt; **25**. Tizi-Ouzou, Makouda, Tigoulmamine.

**Figures 26–28.**
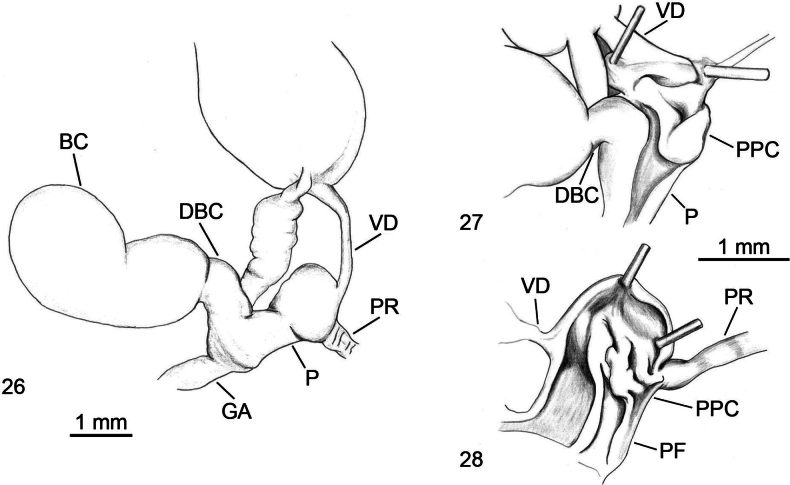
*Ambigolimax parvipenis* genitalia. **26**. Distal genitalia, Algeria, Tigoulmamine; **27**. Internal view of penis, same specimen as in Fig. 26; **28**. Internal view of penis, Algeria, Tizi-Ouzou, Makouda, Tigoulmamine.

#### Diagnosis.

*Ambigolimax parvipenis* is indistinguishable from *A. waterstoni* by external morphology. However, the Algerian specimens show a colouration that is more variable, dark brown specimens with barely visible bands on the mantle, greyish specimens with eight rows of whitish spots on the body plus two dark grey bands on the mantle, and milky white specimens with two pale grey bands along the body (Figs 21–25). Short penis, with a rounded proximal part and a cylindrical and narrower distal part; the vas deferens enters the penial apex, while the retractor muscle is attached at a small distance or more laterally on the proximal penis (Fig. 26). Within the proximal penis, there is a convoluted crest, which in some specimens appears as a thick fleshy structure (Fig. 27); from it, two longitudinal flaps extend straight into the distal penis, joining together at their anterior ends (Fig. 28). This structure shows remarkable variability, the crest inside the penis may be barely outlined. The two parallel flaps may not join in the anterior part of the penis. Bursa copulatrix is variable in shape and size; however, when it is well developed, the bursa copulatrix plus its duct are twice as long as the penis. The long rectal caecum does not reach the posterior tip of the visceral sac.

#### Distribution.

*Ambigolimax parvipenis* is an invasive species with an unknown native range. Its presence in North Africa was first reported by Borredà and Martínez-Ortí (2017), under the name *Lehmannia nyctelia* (see Hutchinson et al. 2022: 31). We document here the first confirmed records of *A. parvipenis* from Algeria, where the species occurs widely in both natural and ruderal habitats, ranging from Algiers to Tizi-Ouzou.

#### Ecology.

Species found mainly under stones and plant debris, sampled in syntopy with *M. nigricans*; it is also found in olive groves and oak forests.

#### Remarks.

Bourguignat (1861: 304) described *Limax raymondianus* based on external characters only: cylindrical body, sharp and briefly keeled back; large, oblong mantle with rounded anterior and posterior margin; dark colour passing towards the foot to a yellowish grey. In the same paper, the caption of fig. 8, plate 16, refers to *Limax raymondianus*; however, this appears to be a misprint as the slug illustrated shows a longer carina and a shield with a furrow, features typical of *Milax*. Bourguignat (1862b) subsequently added two new figures (figs 1, 2, pl. 2) to the original one (now fig. 8, pl. 13) which, instead, correspond well to the description of *L. raymondianus*.

**Figures 29, 30.**
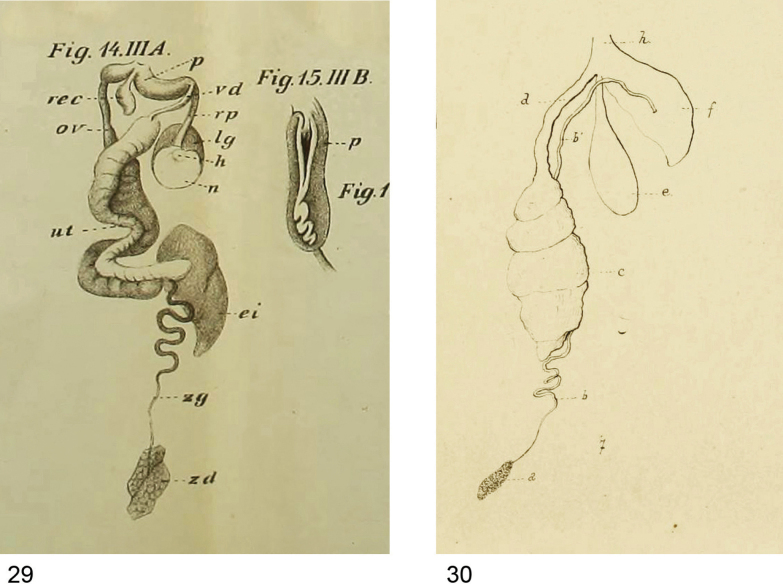
Genital morphology of North African slugs as illustrated in 19^th^ century literature. **29**. Detail of plate 8 from Simroth (1885) showing the genitalia (on the left) and the opened penis (on the right) of the slug named “*Limax nyctelius*”; **30**. Detail of plate 22 from Germain (1908) showing the genitalia of Agriolimax (Malacolimax) kervillei, here regarded as *Ambigolimax* sp.

Simroth (1885: 212, pl. 8, figs 14A, 15A) examined an adult slug from Algeria, showing a reddish-brown colouration on top, with paler flanks and dark chestnut bands on the mantle and back, a short penis, without appendix, and inside a convoluted crest into the proximal part and straight into the distal one (Fig. 29). That would all be consistent with a species of *Ambigolimax*. However, Simroth (1885) noted the absence of a rectal caecum, as in *Malacolimax*. Simroth (1885) misclassified this slug as *L. nyctelius*. Pollonera (1890a) examined some slugs from Oran (northwestern Algeria), lacking a rectal caecum, and he classified them as *Malacolimax raymondianus*. Pollonera described these specimens as having cylindrical bodies, short keels, and a rounded mantle at the front and rear; dark colour, laterally pale; short, thick penis without appendix; short vas deferens. Thus, Pollonera’s *M. raymondianus* corresponds well both to Bourguignat’s original description of *L. raymondianus* and, concerning the anatomical features, to the specimens examined by Simroth (1885). Hutchinson et al. (2022) highlighted the similarity in genital morphology between *A. parvipenis* and the specimens examined by Simroth (1885) and Pollonera (1890a). Nevertheless, Hutchinson et al. (2022) suggested that *L. nyctelius* in Simroth (1885) and *M. raymondianus* in Pollonera (1890a) could be “a species of *Malacolimax* but perhaps of some other unknown or unexpected limacid genus lacking a caecum, or possibly even an aberrant member of a genus whose other species do have a caecum”.

From Algiers, the type locality of *Limax raymondianus*, we collected only one species of *Ambigolimax*, namely *A. parvipenis*. This species appears to be native, as it is widespread in Algeria in natural habitats, including Kabylia and Tizi-Ouzou. If the absence of a caecum in the specimens examined by Simroth (1885) and Pollonera (1890a) was due to an oversight or a malformation, *A. parvipenis* could potentially be considered a junior synonym of *A. raymondianus*. To address this taxonomic issue, we searched for syntypes of *L. raymondianus* in the Bourguignat collection at the Natural History Museum of Geneva (NHMG) and at the National Museum of Natural History in Paris (NHMP). Unfortunately, no syntypes were located. Additional research at the Natural History Museum in London (NHML) also failed to yield syntypes or topotypes. We were able to trace only two specimens from the Bourguignat collection labelled *L. raymondianus*. These cannot be regarded as syntypes, as they originate from Theniet El Had, in the Ouarsenis Massif of the Atlas Tellien, ca 140 km southwest of the type locality, Algiers. One specimen had already been dissected, and its poor state of preservation prevents reliable identification; therefore, these specimens did not provide useful information.

Since the occurrence of a slug genus lacking a caecum in Algeria, as well as the possible presence of additional *Ambigolimax* species near Algiers, cannot be ruled out, we prefer to retain *L. raymondianus* as a taxon inquirendum pending further research.

Germain (1908) described the new species Agriolimax (Malacolimax) kervillei from Aïn-Drahm (Kroumirie, northwestern Tunisia). He illustrated the genitalia of a specimen with a short, thick penis without an appendix, and he stated these slugs were immature. Regteren Altena (1966) pointed out that the reproductive organs figured in the original description of A. (M.) kervillei do not belong to a juvenile specimen as Germain (1908: 141) thought, because the glandular tissue of the sperm oviduct is as well developed as the bursa copulatrix (Fig. 30). We agree with this view. On the other hand, Regteren Altena (1966) speculated that the penis could be strongly contracted and bent backwards. Based on the data presented here, kervillei could be a senior synonym of *A. parvipenis*. However, the schematic drawing provided by Germain (1908) (Fig. 30) shows a penis proportionally larger than that of *A. parvipenis* and the vas deferens attached laterally (apically in *A. parvipenis*). Further studies on specimens from Tunisia are needed to ascertain the taxonomic status of Germain’s species.

Abbes et al. (2010) did not record *Malacolimax* or *Ambigolimax* without a penial appendix from Tunisia, but recorded *L. melitensis* and *L. marginata*. The report of the latter species could be a misidentification of *A. valentianus* (see above and Borredà and Martínez-Ortí 2017).

##### Family Agriolimacidae H. Wagner, 1935

**Subfamily Agriolimacinae H. Wagner, 1935**

### Genus Deroceras

Taxon classificationAnimaliaStylommatophoraAgriolimacidae

Rafinesque, 1820

66B71B5C-07FC-569B-8967-FEC53D26D1F2

#### Diagnosis.

Body length in the Algerian species is 25–40 mm, width 4–6 mm, mantle length 8–15 mm; mantle with pear-shaped outline, anteriorly narrower; pneumostome in posterior position; flattened and truncated rear end of the body; short keel; shell is an oval plate, slightly convex dorsally and inconspicuously concave ventrally, its nucleus is in the posterior left margin, upper surface with concentric irregular growth lines; sack-shaped penis, often irregular or laterally constricted by an incision; with a few exception the penis has external accessory organs: penial gland and appendices, pockets or swellings; inside the penis a variously formed stimulator and folds.

### Deroceras cf. brondelianum

Taxon classificationAnimaliaStylommatophoraAgriolimacidae

(Bourguignat, 1861), comb. nov.

A543A27F-0BC2-5B69-A596-9B4E648C8D83

Figs 31–36, 61

Limax brondelianus – Bourguignat 1861: 302–303 – Environs d’Alger. Bourguignat 1862b: 37–38, pl. 2, figs 5–7 – Environs d’Alger.
Krynickillus brondelianus – Bourguignat 1864: 43–44, pl. 1, figs 9–11 – Environs d’Alger.

#### Material examined.

• 2 exx; Boumerdès, Afir; 36°54.033'N, 3°58.350'E; 10 m a.s.l.; January 2019; R. Ramdini leg. • 3 exx; Tizi-Ouzou, Makouda, Tazarourt; 36°48.083'N, 4°1.367'E; 340 m a.s.l.; November 2020; R. Ramdini leg. • 4 exx; Tizi-Ouzou, Makouda, Tigoulmamine; 36°48.167'N, 4°1.867'E; 350 m a.s.l.; December 2020; R. Ramdini leg. • 5 exx; Tizi-Ouzou, Yakouren, Yakouren Forest; 36°44.750'N, 4°26.100'E; 720 m a.s.l.; December 2020; R. Ramdini leg. • 2 exx; Jijel, National Park of Taza, Garouche forest; 36°40.033'N, 5°38.367'E; 700 m a.s.l.; January 2021; G. Sadouk leg.

**Figures 31–36.**
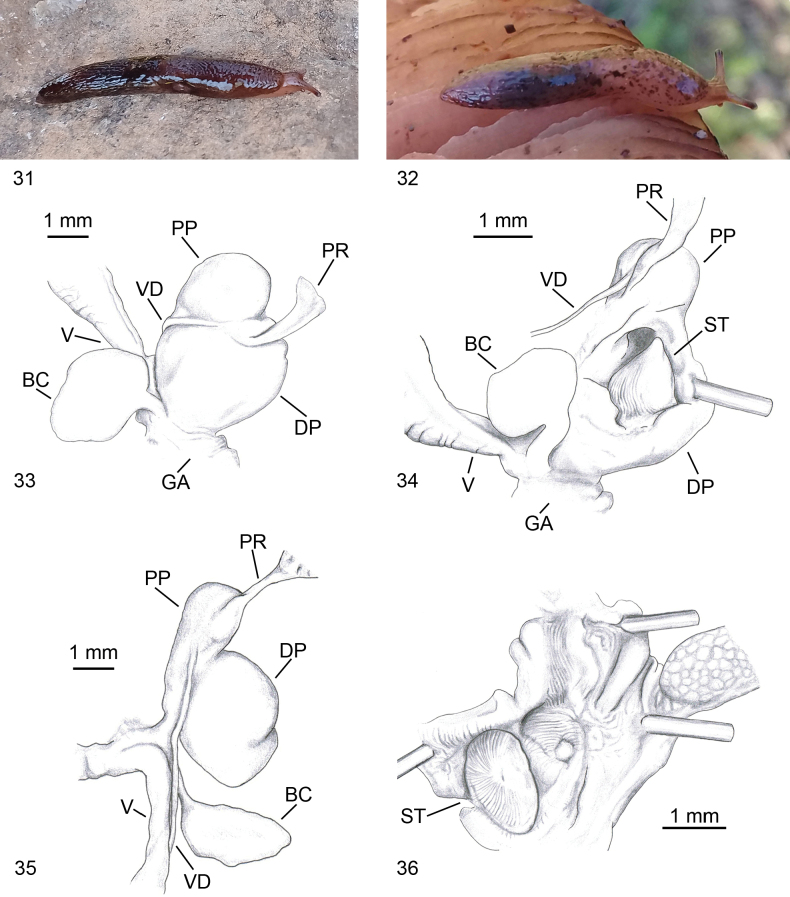
*Deroceras cf. brondelianum*. **31, 32**. Living specimen, Tizi-Ouzou, Makouda, Tigoulmamine; **33**. Genitalia, Tizi Ouzou, Yakouren Forest; **34**. Open genitalia with stimulator, same specimen as in Fig. 33; **35**. Genitalia, Tizi-Ouzou, Makouda, Tigoulmamine; **36**. Internal view of penis, Tizi Ouzou, Yakouren Fores.

#### Diagnosis.

*Deroceras cf. brondelianum* is externally similar to all other *Deroceras* species. Its colour is from dark brown to pale brown to white, with numerous small darker spots (Figs 31–32). Penis divided by a more or less shallow constriction into a broad, rounded, or oval distal part and a smaller, rounded, or oval proximal part; the latter is devoid of appendix and digitiform glands. The vas deferens enters the penis in the transition zone between the distal and proximal penis; the unbranched retractor muscle attaches to the penis side by side with the vas deferens or laterally to the proximal penis (Figs 33–35). Inside the distal penis, there is a cone-shaped stimulator with an oval base (Fig. 34). The stimulator and internal walls of the penis are covered with striae resembling fingerprints (Fig. 36). No caecum was observed.

#### Distribution.

Endemic species of northern Algeria.

#### Ecology.

This slug prefers shady and very humid environments; it has been found only in the winter season (December–February); during the day, it takes refuge under tree trunks and decaying vegetation.

#### Remarks.

In our opinion, *L. brondelianus* should be placed within the family Agriolimacidae based on Bourguignat’s statement that it is closely related to the genus *Krynickia* [synonym of *Krynickillus* Kaleniczenko, 1851 (Agriolimacidae)] as well as on Bourguignat’s description: the respiratory pore located posteriorly on the right margin of the mantle, the size (25 mm contracted, 40–45 mm extended), the broad and anteriorly elongated mantle, and the dorsum briefly keeled.

Bourguignat (1861) described *D. brondelianum* from the surroundings of Algiers as having a very dark back and mantle and yellowish-grey sides. We have examined *Deroceras* specimens from the Boumerdès (ca 40 km east of Algiers) and Tizi-Ouzou regions. These *Deroceras* have a variable external colouration, but some specimens show a colouration similar to that described by Bourguignat for *D. brondelianum*. They may correspond to *Deroceras brondelianum* in our opinion; however, we did not observe the distinctive papillate texture on the tentacles as described by Bourguignat (1861). Whether this papillate texture represents a diagnostic feature of *D. brondelianum*, an indication of an unknown genus, or a malformation caused by infection remains uncertain. In an effort to resolve this taxonomic issue, we searched for syntypes of both *D. brondelianum* and the second *Deroceras* species described by Bourguignat from Algeria, namely *D. subsaxanum* (type locality: Constantina). Unfortunately, no syntypes were located in the Bourguignat collection at NHMG or NHMP. Pending further research on topotypes of *D. subsaxanum* and on the nature of the papillate texture on the tentacles of *D. brondelianum*, we provisionally regard our specimens from Algiers as *Deroceras cf. brondelianum*.

The genitalia of these specimens exhibit characters not known in other *Deroceras* species. *Deroceras neuteboomi* (t.l .: Rhodes Island) is the only recognised species of *Deroceras* s. str. lacking an appendix and a penial gland; however, the *D. cf. brondelianum* differs by the absence of a wrinkled area on the proximal penis (present in *D. neuteboomi*), by the presence of a constriction between the distal and proximal penis, and by the insertions of the retractor muscle and vas deferens located near the penial constriction (more apically in *D. neuteboomi*). Based on these observations, we propose *D. cf. brondelianum* Bourguignat (1861) as a valid species of the genus *Deroceras* s. str., endemic to northern Algeria.

### Deroceras riedelianum

Taxon classificationAnimaliaStylommatophoraAgriolimacidae

Wiktor, 1983

FA61E1D7-B77C-566A-8FC7-319006D9ABA2

Fig. 62

Deroceras riedelianum – Wiktor 1983a: 162–164, figs 14–21 – Algeria: Skikda (= Philippeville); Djebel Edough near Annaba (= Bône) between Seraïdi (= Bugeaud) and Sainte-Croix-d’Edough; Annaba (= Bône) ruins of Roman town Hippona; between Skikda (= Philippeville) and Stora. Wiktor 2000: 368, 393, 500, 502, 511–512, 518–519, 580–582 figs 581–588 – Algeria: Skikda (=Philippeville) and Annaba (= Bône).

#### Material examined.

No specimens in our samples.

#### Diagnosis.

Body length ≤ 23 mm. Colouration pale coffee with dark spotting on the mantle and posterior body. Mantle is attached distally, allowing its anterior portion to be bent upward at ~ 2/3 of its length. Elongated penis, medially constricted; enlarged distal part; proximal part equipped with one smooth process which probably constitutes the penial gland; on the opposite side, there is an appendix-like distension. The vas deferens opens laterally between the process and the distension. The musculus retractor penis is attached between the process and the distension, but apically. The stimulator is cone-shaped, connected with a big fold running toward the proximal penis.

#### Distribution.

So far, known only from the type locality: surroundings of Skikda and Annaba (northwestern Algeria).

#### Ecology.

This species has been found in brushwood, cork oak forests, ruins, and rock piles; it takes refuge in wet litter and under tree trunks (Wiktor 2000).

#### Remarks.

Bourguignat (1862b) described *Limax subsaxanus* as characterised by a stocky anterior body and a sharp and slender back, pink body, and white foot, similar to the genus *Krynickia* (= *Krynickillus* (Agriolimacidae)) due to the mantle being large and much detached in its anterior portion. The description in Bourguignat (1861) and the figure of the shell and body in Bourguignat (1864: pl. 3, figs 13–16) allow us to attribute *L. subsaxanus* to the genus *Deroceras*.

*Deroceras subsaxanum*, described from Constantina (ca 85 km southwest of Annaba and Stora), may be a senior synonym for *D. riedelianum*. However, the relationship of *D. subsaxanum* to both *D. riedelianum* and *D. cf. brondelianum* can only be clarified by the study of topotypical specimens.

Castillejo (1996, 1997) and Borredà and Martínez-Ortí (2017, 2022) considered *D. riedelianum* a junior synonym of *D. ponsonbyi* (Hesse, 1884). Following Wiktor (2000), we disagree with this view: Wiktor (2000) highlighted that *D. ponsonbyi* has a bifurcated penial retractor muscle, with one insertion in the distal penis and the other one in the proximal penis, while *D. riedelianum* has only one insertion in the proximal penis; secondly, the penial appendix appears different in the two species. In addition, *D. ponsonbyi* shows glandular tissue covering the distal penis distention (absent in *D. riedelianum*). We regard *D. ponsonbyi*, known from the Gibraltar area, as an endemic species to southern Spain and *D. riedelianum* as endemic to northeastern Algeria (Kabylia). The *Deroceras* species recorded (under *D. agreste*) by several nineteenth-century authors in western Algeria (Oran) and northern Morocco require further research (Terver 1839; Morelet 1853; Hesse 1884; Heynemann 1885; Pollonera 1891).

##### Family Vitrinidae Fitzinger, 1833

**Subfamily Vitrininae Fitzinger, 1833**

**Genus *Vitrina* Draparnaud, 1801**

### ? Vitrina letourneuxi

Taxon classificationAnimaliaStylommatophoraVitrinidae

(Bourguignat, 1864)

26A87517-590A-59C4-A021-590700F997C0

Figs 72, 73

Vitrina letourneuxi – Bourguignat 1864: 303 – Sommet du petit atlas de Blida, près du marabout de Sidi-Abd-el-Kader. Sturany 1909: 292 – Algeria.
Vitrina? letourneuxi – Forcart 1959: pl. 1, fig. 1 (Lectotype) – Kleiner Atlas von Blida beim Marabout Sidi-Abd-el-Kader, 1600 m.

#### Material examined.

No specimens in our samples.

**Figures 37–39.**
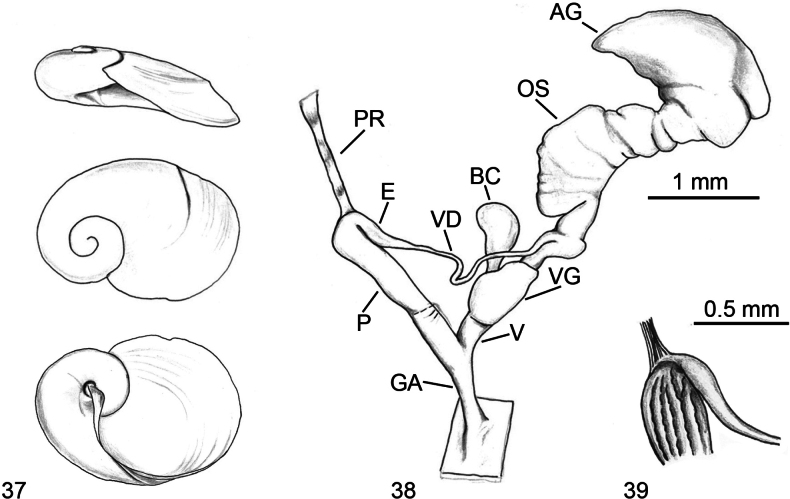
*Daudebardia brevipes letourneuxi*, Algeria, Jijel, National Park of Taza, Garouche forest. **37**. Shell; **38**. Genitalia; **39**. Internal view of the proximal penis.

**Figures 40–45.**
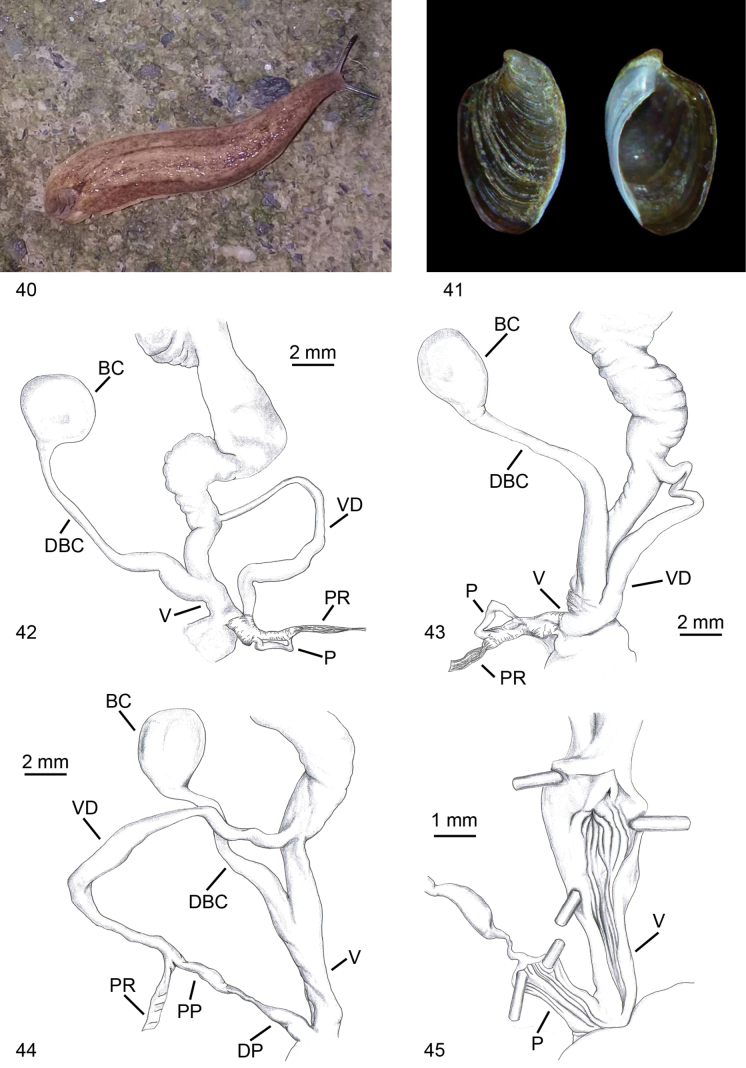
*Testacella riedeli*, Algeria Tizi-Ouzou, Makouda, Tigoulmamine. **40**. Living specimen; **41**. Shell; **42**. Distal genitalia. **43–45**. Distal genitalia of a second specimen; **43**. Distal genitalia; **44**. Details of distal genitalia; penis without the thick glandular and muscular sheath; **45**. Internal view of penis and vagina.

**Figures 46–71.**
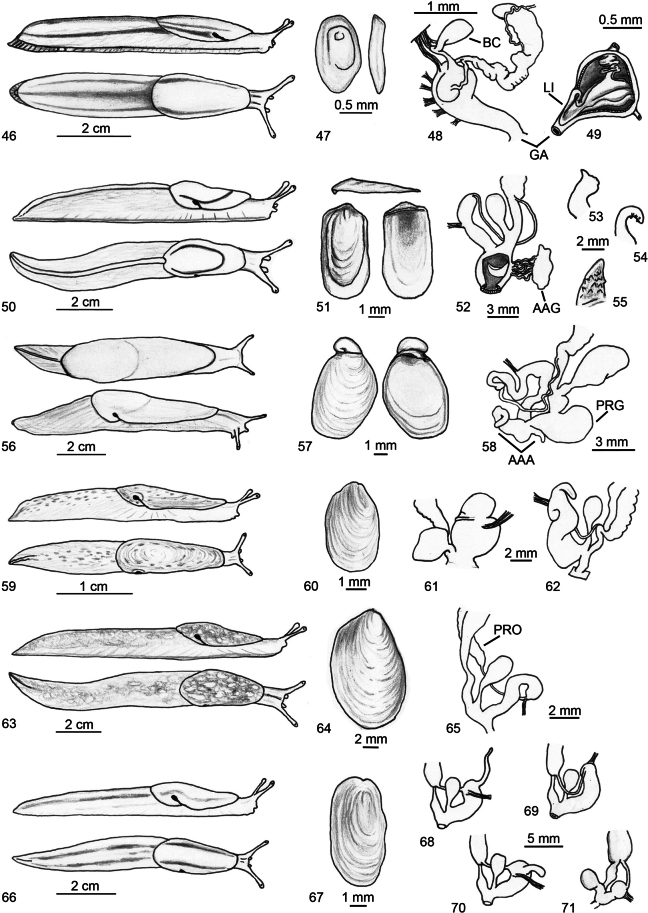
Identification key of Algerian slugs. **46–49**. *Letourneuxia numidica* from Wiktor (1983a) modified; **46**. Mollusc in lateral and dorsal view; **47**. Shell in dorsal and lateral view; **48**. Genitalia; **49**. Atrial ligula. **50–55**. Genus *Milax*; **50**. Mollusc in lateral and dorsal view; **51**. Shell in lateral, dorsal and ventral view; **52**. Schematic drawing of genitalia, with a section of the atrium and stimulator; **53**. Stimulator of *M. ater*; **54**. Stimulator of *M. gagates*; **55**. Stimulator of *M. nigricans*. **56–58**. *Drusia deshayesii*; **56**. Mollusc in lateral and dorsal view; **57**. Shell in dorsal and ventral view; **58**. Distal genitalia. **59–62**. Genus *Deroceras*; **59**. Mollusc in lateral and dorsal view; **60**. Shell in dorsal view; **61**. Distal genitalia of *D. cf. brondelianum*; **62**. Distal genitalia of *D. riedelianum* from Wiktor (1983a) modified. **63–65**. *Limacus flavus*; **63**. Mollusc in lateral and dorsal view; **64**. Shell in dorsal view; **65**. Distal genitalia. **66–71**. Genus *Ambigolimax*; **66**. Mollusc in lateral and dorsal view; **67**. Shell in dorsal view; **68**. Distal genitalia of *A. melitensis*; **69**. Distal genitalia of *A. waterstoni*; **70**. Distal genitalia of *A. valentianus*; **71**. Distal genitalia of *A. parvipenis*.

**Figures 72–82.**
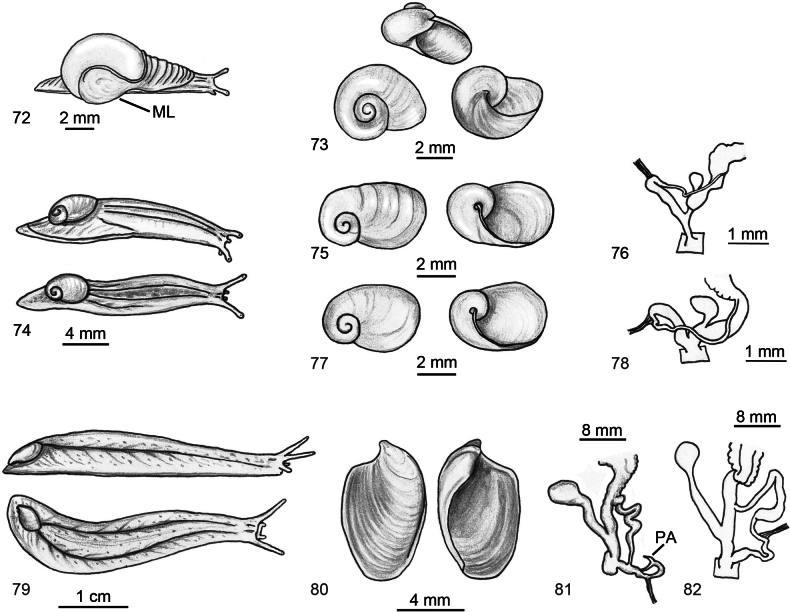
Identification key of Algerian semi-slugs. **72, 73**. Genus *?Vitrina*; **72**. Schematic drawing of the mollusc; **73**. Shell in frontal, apical and ventral view. **74–78**. Genus *Daudebardia*; **74**. Mollusc in lateral and dorsal view; **75**. Shell in dorsal and ventral view of *D. brevipes letourneuxi*; **76**. Distal genitalia of *D. brevipes letourneuxi*; **77**. Shell in dorsal and ventral view of *D. rufa atlantica*; **78**. Distal genitalia of *D. rufa atlantica* from Riedel (1978) modified. **79–82**. Genus *Testacella*; **79**. Mollusc in lateral and dorsal view; **80**. Shell in dorsal and ventral view; **81**. Distal genitalia of *T. fischeriana*; **82**. Distal genitalia of *T. riedeli*.

#### Diagnosis.

Shell vitrinid-like, apex slightly immersed, spire with 3 1/2 convex whorls; the first 2 1/2–3 whorls with a microsculpture of spirally arranged pits; radial striae near the suture and irregularly subplicate last whorl; transversely oblong aperture; peristome with rounded basal margin and columellar margin folded on the umbilicus; shell maximum height (H): 3 mm, shell maximum diameter (D): 5 mm. Based on Bourguignat’s original description, the mollusc can withdraw completely into the shell; it has a transparent, greyish-white body; the mantle is darker at the edges and covers most of the spire; the large tentacles are brown-violet; the rear part of the body is pointed and slightly brownish. Genitalia Unknown.

#### Distribution.

So far known only from the type locality: at the top of the small atlas of Blidah, near the marabout of Sidi-Abd-el-Kader.

#### Ecology.

*?Vitrina letourneuxi* lives under moist debris of shavings, branches, and needles of *Cedrus atlantica* ((Endl.) Manetti ex Carrière, 1855).

#### Remarks.

Sturany (1909) hypothesised a close relationship between *?V. letourneuxi* and the endemic *Oligolimax* species from Libya. Forcart (1959) rejected this hypothesis and suggested that the Algerian species belongs to the genus *Vitrina*. In our opinion, based on the descriptions of Bourguignat (1864) and Forcart (1959) and its geographical isolation, it cannot be excluded that ?*V. letourneuxi* belongs to other unknown or unexpected vitrinid genera. This question can only be resolved by studying genital morphology and/or by molecular analysis.

##### Superfamily Gastrodontoidea Tryon, 1866

**Family Oxychilidae P. Hesse, 1927 (1879)**

**Subfamily Daudebardiinae Kobelt, 1906**

**Genus *Daudebardia* W. Hartmann, 1821**

### Subgenus Daudebardia

Taxon classificationAnimaliaStylommatophoraVitrinidae

W. Hartmann, 1821

8469477C-BADE-5296-8408-78F08BEB0DBE

#### Diagnosis.

Body in adult specimens (17–20 mm) much broader and longer than the shell (4–5 mm); with two longitudinal furrows on the back and less distinct, oblique furrow on each side; bluish-grey in colour; tripartite whitish sole; shell small, external, umbilicated, shining, transparent; paucispiral, the last whorls flaring strongly in adult; genitalia characterised by cylindrical penis and thin, short epiphallus which passes insensibly to the vas deferens; penial retractor muscle attached at the junction between penis and the epiphallus; vagina and duct of the bursa copulatrix partially covered by a vaginal gland.

### Daudebardia (Daudebardia) brevipes letourneuxi

Taxon classificationAnimaliaStylommatophoraVitrinidae

Bourguignat, 1866

54E82D9D-90E9-522D-AC41-EBE041DA64AA

Figs 37–39, 75, 76

Daudebardia letourneuxi – Bourguignat 1866; p. 210, pl. 33, figs 7–12 – Forêt de l’Édough, près de Bône. Letourneux 1870: 278 – Bone, Saint-Croix-de-l’Édough, forêt de l’Édough.
Daudebardia platystoma – Letourneux 1870: 268, 291–292 – Algeria, Kabylia: Tizi-n-Cheriâ, col d’Akfadou, source Tala Guizan; Soummes.
Daudebardia charopia – Letourneux 1870: 278, 292–293 – Bone, Saint-Croix-de-l’Édough, forêt de l’Édough; prés de Bone.
Daudebardia (Daudebardia) brevipes letourneuxi – Riedel 1978: 148, figs 12–17 – Bekannt von der Umgebung von Annaba (u. a. Djebel Edough) und von Kabylien – die Quelle Tala-Guizan in Col d’Akfadou (ca. 30 km östlich Fort-National).

#### Material examined.

• 2 exx; Jijel, National Park of Taza, Garouche forest; 36°40.033'N, 5°38.367'E; 700 m a.s.l.; January 2021; G. Sadouk leg.

#### Diagnosis.

The shell is similar to the topotypical specimens depicted by Riedel (1978: 146, figs 12–14) in which the columellar margin covers part of the umbilicus (Fig. 37); H: 7.4 mm × D: 5.2 mm; H: 6.9 mm × D: 4.8 mm; genitalia characterised by a cylindrical penis, slightly wider apically; thin and short epiphallus, which passes insensibly to the vas deferens; vagina as long as half penis and covered for three-quarters of its length by a vaginal gland; duct of the bursa copulatrix emerging from the vaginal gland and ending in an oval bursa copulatrix; atrium as long as the vagina (Fig. 38); internal walls of the penis with four thin crests, two of which distally merge into a single crest (Fig. 39); internal walls of the vagina with an irregular and thin crest.

#### Distribution.

Currently known from northeastern Algeria.

#### Ecology.

It lives in humid litter among leaves and plant debris or under stones, feeding on invertebrates.

#### Remarks.

The genus *Daudebardia* was recorded from Algeria (Kabylia region) by Bourguignat (1866, 1870) and Letourneux (1870, 1872). These authors introduced five new nominal species. *Daudebardia letourneuxi* Bourguignat, 1866 (t.l. Bona = Annaba, northeastern Algeria) is the oldest name for the genus *Daudebardia* in Algeria. It was described based on an immature shell (Bourguignat 1866). Riedel (1978) re-examined the holotype and a topotypical adult shell from the Hagenmuller collection (ex-Letourneux coll.) and tentatively classified this taxon as a subspecies of *D. brevipes*. However, the shells do not show relevant differential characters from the nominotypical subspecies. Riedel (1978) selected lectotypes of *D. charopia* Letourneux, 1870 (t.l. Bona) and *D. platystoma* Letourneux, 1870 (t.l. Tala Guizan en Kabylie) from topotypical specimens preserved in the Bourguignat collection and proposed these two taxa as junior synonyms of *D. brevipes letourneuxi*. The two specimens from Jijel we examined (Fig. 38) show the atrium as long as the vagina; this feature is not known for other *Daudebardia* species.

### Daudebardia (Daudebardia) rufa atlantica

Taxon classificationAnimaliaStylommatophoraVitrinidae

Bourguignat, 1870

0AD7CBFC-07FB-52C8-8AB7-51D45DF812A7

Figs 77, 78

Daudebardia atlantica – Bourguignat 1870, p. 15–16, pl. 4, figs 9–12 Moll. nouv. 2 – Dans la forêt de l’Edough, près de Bone.
Daudebardia (Daudebardia) rufa atlantica – Wagner 1952: 122–123 – In dem Wald von Edough bei Bône in Algerien und Tala-Guizaa, Kabylia. Riedel 1978: 145–148, figs 8–9, 18–19 – Lebt in der Umgebung von Annaba in NO-Algerien, hauptsächlich im Gebirge Djebel Edough.

#### Material examined.

No specimens in our samples.

#### Diagnosis.

The shell is oval, the coiled part of the shell occupies 40–50% of the length (due to the less narrowly coiled spire), and the umbilicus is moderately wide. It differs from *Daudebardia brevipes* by its regularly rounded last whorl (straight in *D. brevipes*), which gives the shell an oval rather than an oblong shape; the initial whorls are less narrowly coiled and occupy a larger portion of the fully grown shell, umbilicus is wider.

#### Distribution.

Northeastern Algeria.

#### Ecology.

It lives in humid litter among leaves and plant debris or under stones, feeding on invertebrates.

#### Remarks.

Riedel (1978: 9, figs 18, 19) re-examined the type series of *D. atlantica* Bourguignat, 1870 (t.l. Bone), selected a lectotype and provided the first description and illustration of the genitals. He tentatively classified *D. atlantica* as a subspecies of *D. rufa*, although the shell and the genitals do not show relevant differential features. Borredà and Martínez-Ortí (2017) listed with a question mark the presence in Algeria of the two most common European species, *D. brevipes* (Draparnaud, 1805) and *D. rufa* (Draparnaud, 1805). Although a modern revision of the genus *Daudebardia* s. str. is urgently needed, we prefer to maintain the classification proposed by Riedel (1978), considering the geographical isolation of the Algerian populations and the few known data points on the genitalia of *D. rufa atlantica*. Riedel (1978) regarded *D. nubigena* Bourguignat, 1870 as a subspecies of *D. rufa*, characterised by a wider last whorl in the spiral part. Based on the original description of *D. nubigena* and the drawing of the syntype illustrated by Riedel (1978: 146, figs 10–11), it could be a junior synonym of *?Vitrina letourneuxi*. The two taxa, *D. nubigena* and ?*V. letourneuxi*, were described from the same locality, i.e., the summit of the Little Atlas near Blida, Algeria.

##### Infraorder Arionoidei

**Subfamily Arionoidea Gray, 1840**

**Family Arionidae Gray, 1840**

### Genus Letourneuxia

Taxon classificationAnimaliaStylommatophoraVitrinidae

Bourguignat, 1866

821C1DA1-3838-5998-8803-1626F02DF9B7

#### Diagnosis.

Pneumostome antemedial, posterior body end rounded with a small caudal mucus gland; genitalia without epiphallus and penis, large atrium with a ligule inside. Monospecific genus.

### Letourneuxia nyctelia

Taxon classificationAnimaliaStylommatophoraVitrinidae

Bourguignat, 1861

0026B30D-7DD7-5416-AF39-7E516A3B1A9D

Figs 46–49

Limax nyctelius – Bourguignat 1861: 305–306 – Environs de Bougie et d’Alger, sur la colline de Budjaria, de Tlemcen, Aïn-el-Haout – et Oran – d’Alger, de Constantine, de Boghar.
Letourneuxia numidica – Bourguignat 1866: 201, pl. 34, figs 1–7 – Tlemcen (NW-Algeria). Wiktor 1983a: 156–160, figs 1–13. – Morocco: Taza-Bab Rih; Ras El Oued, 12 km south of Taza; Beni Hozmar Mts., 3–5 km south of Tetuan.
Letourneuxia atlantica – Bourguignat in Pechaud 1884: 6 – Tlemcen (NW-Algeria).
Arion (Ariunculus) moreleti – Hesse 1884: 14, pl. 8, figs 1–4 – Tanger (Morocco).
Geomalacus (Letourneuxia) turneri – Pollonera 1890b: 38 – Oran (Algeria).
Ariunculus pallaryi – Collinge 1904: 47 – Echmühl, Oran, Algeria. syn. nov.
Geomalacus (Letourneuxia) maroccanus – Pollonera 1916: 191 – Grand Atlas (= Haut Atlas, Morocco).
Letourneuxia nyctelia – Hutchinson et al. 2022: 19–41 – Algeria, Morocco.

#### Material examined.

No specimens in our samples.

#### Diagnosis.

Mantle narrowed in front, widely rounded backwards, pneumostome antemedial; body rear blunted, with caudal mucus gland weakly developed; body reddish-brown with a dark-brown back and lateral band, reddish mantle with a lateral dark-brown band; thick pentagonal shell with an eccentric nucleus, without growth striae; genitalia characterised by: oviduct tube-shaped, opening to atrium slightly laterally, without bulbus; large atrium with a large ligule inside; without penis and epiphallus; however, Pollonera (1890b) referred to a small penis which gradually passes to the vas deferens; bifurcated genital retractor muscle, one branch attached to the duct of the bursa copulatrix, the other branch to the posterior part of the atrium (Wiktor 1983a).

#### Distribution.

Described from Tlemcen, Oran, northwestern Algeria, it is also recorded from northeastern Morocco.

#### Ecology.

*Letourneuxia nyctelia* lives on rocks near streams, among herbs, lime debris, oleander scrubs or pine-oak forest, pine trees, and eucalyptus trees.

#### Remarks.

For many decades, this species was known under the name *L. numidica*, but Hutchinson et al. (2022) recently proposed *nyctelia* as its senior name. Collinge (1904) described the external features and genitalia of a new species, *Ariunculus pallaryi* Collinge, 1904 which correspond well to *L. nyctelia*. The type locality of *A. pallaryi* is Echmühl, situated in the same Algerian region where *L. nyctelia* is widespread (Oran, western Algeria).

*Ariunculus pallaryi* Collinge, 1904 (t.l. Echmühl, Oran, Algeria) is here proposed as a new junior synonym of *L. nyctelia* Bourguignat, 1861, following the suggestion of Hutchinson and Reise (2015).

Aucapitaine (1862) reported specimens of *Arion rufus* from Kabylia, differing from European individuals only in their smaller size. No later author has confirmed the presence of arionids in Kabylia.

##### Infraorder Helicina

**Superfamily Testacelloidea Gray, 1840**

**Family Testacellidae Gray, 1840**

**Genus *Testacella* Lamarck, 1801**

### Subgenus Testacella

Taxon classificationAnimaliaStylommatophoraVitrinidae

Lamarck, 1801

97C07CC6-5DDB-5D30-88F9-B3D2278A3B92

#### Diagnosis.

Body wide, rather flat (length ≤ 45 mm, width 10–15 mm) with two evident dorsal grooves, orange-brown (Fig. 40); shell *Haliotis*-like, reduced, and rather thick, external, and situated near the posterior end of the body (Fig. 41).

### Testacella (Testacella) fischeriana

Taxon classificationAnimaliaStylommatophoraVitrinidae

Bourguignat, 1862

CD6574CB-1E6E-5978-815B-5DAD2C5AE17F

Fig. 81

Testacella fischeriana – Bourguignat 1862c: 516–517, pl. 13, figs 5–7 – Environs de Constantine, d’Alger. Giusti et al. 1995: 320, figs 300–301, 303–304 – Sidi Kouzeil, Mount Edough, Annaba, Algeria; Gorges du Rhumel, Costantine, Algeria.
Testacella brondeli – Bourguignat 1862b: 521–522, pl. 13, figs 14–16 – Environs de Bone, en Algérie.

#### Material examined.

No specimens in our samples.

#### Diagnosis.

Enlarged vas deferens; penis slender, partially enveloped in a muscular-glandular sheath from which the penial retractor emerges; a penial diverticulum originating at the point where the proximal penis ends, and the distal penis begins.

#### Distribution.

Northeastern Algeria and northern Tunisia (Giusti et al. 1995; Abbes et al. 2010).

#### Ecology.

*Testacella fischeriana* live in earthworm burrows and soil crevices, hunting and eating earthworms.

#### Remarks.

*Testacella fischeriana* is a well-known species. Giusti et al. (1995) recorded it from Annaba, Sidi Kouzeil, and Constantine.

### Testacella (Testacella) riedeli

Taxon classificationAnimaliaStylommatophoraVitrinidae

Giusti, Manganelli & Schembri, 1995

6ED0D8E8-3F96-5A9A-9D05-E1E889EB3EB0

Figs 40–45, 82

Testacella riedeli – Giusti et al. 1995: 317–326, figs 288–293, 302 – Col de Kefrida, Bejaia, Algeria.

#### Material examined.

• 2 exx; Algiers, Dely Ibrahim; 36°45.233'N, 2°59.117'E; 260 m a.s.l.; October 2018; R. Radmini leg. (Figs 40, 41). • 3 exx; Tizi-Ouzou, Makouda, Tigoulmamine; 36°48.167'N, 4°1.867'E; 350 m a.s.l.; November 2019; R. Radmini leg. • 5 exx; Tizi-Ouzou, Yakouren, Yakouren Forest; 36°44.750'N, 4°26.100'E; 720 m a.s.l.; November 2019; R. Radmini leg. • 3 exx; Tizi-Ouzou, Mizrana, Mizrana Forest; 36°50.050'N, 4°4.533'E; 730 m a.s.l.; November 2019; R. Radmini leg. • 2 exx; Jijel, National Park of Taza, Garouche forest; 36°40.033'N, 5°38.367'E; 700 m a.s.l.; January 2021; G. Sadouk leg. • 1 ex; Jijel, National Park of Taza, Tabola; 36°42.567'N, 5°33.000'E; 54 m a.s.l.; January 2021; G. Sadouk leg.

#### Diagnosis.

Vas deferens long, distally widened and with irregularly spaced muscles; penis enveloped by a thick muscular and glandular sheath, from which penial retractor muscle emerges (Figs 42, 43); proximal penis long and slender; distal penis half length of proximal penis, progressively widening before entering genital atrium (Fig. 44); short wide vagina; initially flared duct of the bursa copulatrix, ending in large oval bursa copulatrix; internal walls of the distal penis and vagina with several longitudinal flaps (Fig. 45).

#### Distribution.

Northern Algeria, where it is widespread and quite common, and Gozo Island (Maltese Islands) (Giusti et al. 1995).

#### Ecology.

Testacellids are subterranean and emerge only on wet days. They feed primarily on earthworms.

#### Remarks.

The populations examined enable us to expand the range of this species to the west, up to the Algiers province (Fig. 1B). Additionally, we provide the first morphological data on the internal structure of the penis and vagina (Fig. 45).

##### Key for the identification of the Algerian slugs and semi-slugs, referring to fully grown, living specimens

**Table:** 

1	Shell internal and placed under the mantle	**2**
–	Shell external and placed near the posterior end of body	**6**
2a	Pneumostome lies in the posterior mantle section, dorsal carina (keel) all along the back, mantle crossed by a groove	**3**
–2b	Body 60–70 mm, pneumostome lies in the posterior mantle section, dorsal carina occupies only the posterior end of the body, mantle without a groove, shell oval with the nucleus in the posterior left margin	**4**
–2c	Pneumostome antemedial, posterior body end rounded (not pointed), without keel; on mantle and back, two lateral dark bands; shell oval, thick, with eccentric nucleus; genitalia without epiphallus and penis, large atrium with a ligula inside	***Letourneuxia nyctelia*** (Figs 46–49)
3a	Body 30–100 mm, back slightly > 1/2 of the body length; mantle crossed by a rhomboidal or horseshoe-like groove; shell nail-like with the apex on the major axis and in ventral view truncated posterior end; genitalia characterised by a mass of atrial accessory glands communicating via multiple ducts with atrium and a stimulator contained inside the genital atrial cavity	***Milax*** (Figs 50–52)
–	Body uniformly black, in some specimens, head and sides downwards lighter; oval-conical smooth stimulator, on its tip, there are small flat processes or a sort of fan; penis roughly equal in length to epiphallus; spermatophore unknown	***Milax ater*** (Fig. 53)
–	Body uniformly blackish or pale greyish; stimulator narrow, tongue-shaped, gradually narrowing towards its end, smooth with few irregularly scattered, small, spiny papillae towards the apex; epiphallus claviform and double penis length; spermatophore elongate, posterior end somewhat wider, spines branching dichotomously yet quite irregularly, covering one side of the spermatophore, the other side is smooth	***Milax gagates*** (Fig. 54)
–	Body with a remarkable variability in the external colouring; stimulator conical with acuminate to rounded papillae randomly distributed on the inner side, most of them situated at the stimulator base and absent at its tip; spermatophore elongate conical, covered on all sides and along its whole length by multiply branched spines	***Milax nigricans*** (Fig. 55)
3b	Body 130–140 mm; back a 1/4 of the body length; mantle 1/2 body length, mantle crossed by a large semicircular shallow groove; shell consists of a short spiral part attached to a flat plate; genitals characterised by 2 accessory atrial appendices slightly different in size, penis with extrusion, epiphallus internally reticulated	***Drusia*** (Figs 56–58)
–	Body uniform red-brown; genitals characterised by a cylindrical penis with a swelling in the middle; short atrium, atrial accessory appendices with a big fold inside, very large perivaginal gland	***Drusia deshayesii*** (Fig. 58)
4a	Body 25–30 mm; posterior end of the body truncated, with a more or less raised keel; mantle ≥ 1/3 of the body length and narrower anteriorly, shell oval, thin, glassy; sack-shaped penis, with a stimulator inside; intestine without caecum or with a short caecum	***Deroceras*** (Figs 59–60)
–	Penis divided by a shallow constriction into a broad oval distal part and a smaller oval proximal part, without appendix and digitiform glands; vas deferens enters the penis in the transition zone between distal and proximal penis; cone-shaped stimulator inside the distal penis	***Deroceras cf. brondelianum*** (Fig. 61)
–	Penis elongated with an enlarged distal part and proximal part equipped with one smooth process and appendix-like distension. Stimulator cone-shaped, connected with a big fold	***Deroceras riedelianum*** (Fig. 62)
4b	Body 40–60 mm; posterior end of body pointed with ill-defined carina; mantle < 1/3 of the whole body length; penis elongate, cylindrical; intestine with long caecum	**5**
5a	Body length ≤ 120 mm, yellowish or greenish brown body with irregular yellowish spots; shell thin, oblong-oval, protoconch not protruding; prostate longer than oviduct and not fused to oviduct at its anterior end (Fig. 65); penis without appendix	***Limacus flavus*** (Figs 63–65)
5b	Body length ≤ 80 mm; shell elongated-oval, with protoconch slightly protruding; prostatic gland fused to oviduct for gland’s entire length; duct of bursa copulatrix connected with penis; penis with or without an undivided appendix, inside the penis one or two flaps	***Ambigolimax*** (Figs 66, 67)
–	Penis sac-like, C-shaped, a long, slender, tapering flagellum entering the penis tip laterally; inside the penis a “S” or “C”-shaped flap	***Ambigolimax melitensis*** (Fig. 68)
–	Penis elongated-cylindrical, often swollen at apex; without appendix; inside the penis, two flaps originate from the penial apex and run parallel	***Ambigolimax waterstoni*** (Fig. 69)
–	Penis claviform, posteriorly broadened, with a short blunt penial appendix; the latter arises laterally from the proximal penis, a V-shaped flap inside the penis	***Ambigolimax valentianus*** (Fig. 70)
–	Short penis compared to the length of bursa + duct, divided into a rounded proximal part and a cylindrical and narrower distal part, without appendix; within the proximal penis, there is a convoluted crest, and from it, 2 longitudinal flaps extend straight into the distal penis. The internal penial structure shows a certain degree of variability	***Ambigolimax parvipenis*** (Fig. 71)
6a	Body 10–15 mm, greyish-white; mantle lobes cover most of the shell spire; shell consists of 3 1/2 convex whorls, the first 2 1/2–3 whorls with a microsculpture of spirally arranged pits and radial striae near the suture, umbilicus closed; genitalia unknown	***?Vitrina letourneuxi*** (Figs 72, 73)
6b	Body 17–20 mm, bluish grey, shell shining, transparent, ombelicated, the last whorls flaring strongly; genitalia characterised by a cylindrical penis, short epiphallus, penial retractor muscle attached at the junction between penis and epiphallus; vagina and duct of the bursa copulatrix partially covered by a vaginal gland	***Daudebardia*** (Fig. 74)
–	Shell oval, the coiled part occupies 30–40% of the length in a fully grown shell, umbilicus narrow; genital atrium as long as the vagina	***D. brevipes letourneuxi*** (Figs 75, 76)
–	Shell oval-oblong, the coiled part of the shell occupies 40–50% of the length (due to the less narrowly coiled spire), umbilicus moderately wide	***D. rufa atlantica*** (Figs 77–78)
6c	Body 35–40 mm, wider at the back, rather flat, orange-brown in colour; shell *Haliotis*-like and rather thick	***Testacella*** (Figs 79–80)
–	Penis slender, a penial diverticulum originating where the proximal penis ends and the distal penis begins	***T. fischeriana*** (Fig. 81)
–	Penis without penial diverticulum: proximal penis long and slender; distal penis half proximal penis length, progressively widening before entering the genital atrium.	***T. riedeli*** (Fig. 82)

## Discussion

Based on our results and those of previous studies, 17 slug and semi-slug species have been recorded in Algeria. Six (sub)species are endemic: *Milax ater*, *Deroceras cf. brondelianum*, *D. riedelianum*, *Daudebardia brevipes letourneuxi*, *D. rufa atlantica*, and *?Vitrina letourneuxi*, while one species is subendemic (i.e., with more than 80% of its distribution in Algeria), *Testacella riedeli*.

*Letourneuxia nyctelia* and *Drusia deshayesii* are Moroccan-Algerian species, while *Testacella fischeriana* is a Tunisian-Algerian species. Six species have vast ranges, extending into Europe: *Milax gagates*, *M. nigricans*, *Ambigolimax melitensis*, *A. parvipenis*, *A. waterstoni*, and *A. valentianus*. These broad distributions are partly the result of anthropogenic dispersal.

*Limacus flavus* is regarded as an alien species introduced into the study area. Although it has been recorded in Algeria and North Africa since the 19^th^ century, its occurrence is restricted to the vicinity of major urban centres. In contrast, the other Algerian slug species exhibit a wider distribution across the Maghreb, including natural habitats.

In Algeria, the range of *L. nyctelia* and *D. deshayesii* is limited to the northwest. In contrast, the number of species occurring exclusively in northeastern Algeria (from Algiers to Annaba) is greater, including *Milax ater*, *M. nigricans*, *Ambigolimax melitensis*, *A. parvipenis*, *A. valentianus*, *A. waterstoni*, *Deroceras cf. brondelianum*, *D. riedelianum*, *Daudebardia brevipes letourneuxi*, *D. rufa atlantica*, *?Vitrina letourneuxi*, *Testacella fischeriana*, and *T. riedeli* (see Table 1). Northeastern Algeria is, therefore, the region with the highest rate of biodiversity and endemism, with 14 species, almost half of which are endemic. There are two main reasons for this. Firstly, the Plio-Pleistocene glaciations repeatedly brought the Maghreb palaeocoast close to the southern European paleocoasts (Sicily and Sardinia to the east), facilitating jump dispersal during sea level lowstands (Flemming et al. 2003). These processes were followed by faunal isolation between Europe and North Africa during interglacial climate cycles, which facilitated the diversification and speciation. Secondly, northeastern Algeria shows an increase in humidity from west to east and a greater diversity of habitat types in the country, ranging from the mountainous regions of the Djurdjura (2300 m) which are often snow-covered during the winter months, to the Mediterranean coastal regions.

**Table 1.** List of species sampled in each locality.

**1**	Algiers	Ain Benian	*M. nigricans*.
**2**	Algiers	Dely Ibrahim	*M. nigricans*, *A. parvipenis*, *T. riedeli*.
**3**	Algiers	Douera	*M. nigricans*.
**4**	Algiers	Sidi Moussa	*M. nigricans*.
**5**	Algiers	Tamentfoust	*M. nigricans*.
**6**	Boumerdès	Afir	*M. nigricans*, *Der. cf. brondelianum*.
**7**	Boumerdès	Chabet El Ameur	*M. nigricans*.
**8**	Boumerdès	Corso	*M. nigricans*, *A. waterstoni*.
**9**	Boumerdès	Issers	*M. nigricans*.
**10**	Jijel	National Park of Taza, Garouche forest	*M. gagates*, *Der. cf. brondelianum*, *Daud. b. letourneuxi*, *T. riedeli*.
**11**	Jijel	National Park of Taza, Tabola	*T. riedeli*.
**12**	Relizane	El Hamadna	*Drusia deshayesii*.
**13**	Tizi-Ouzou	Ain El Hammam	*M. gagates*.
**14**	Tizi-Ouzou	Akvil, Ait Ouavan Forest	*M. ater*, *A. waterstoni*.
**15**	Tizi-Ouzou	El Kahra	*M. gagates*.
**16**	Tizi-Ouzou	Makouda, Tazarourt	*M. gagates*, *M. nigricans*, *Der. cf. brondelianum*.
**17**	Tizi-Ouzou	Makouda, Tigoulmamine	*M. nigricans*, *A. parvipenis*, *Der. cf. brondelianum*, *T. riedeli*.
**18**	Tizi-Ouzou	Mizrana, Mizrana Forest	*M. nigricans*, *A. parvipenis*, *T. riedeli*.
**19**	Tizi-Ouzou	Yakouren, Yakouren Forest	*M. nigricans*, *A. parvipenis*, *Der. cf. brondelianum*, *T. riedeli*.
**20**	Tizi-Ouzou	Ouadhias	*M. nigricans*.
**21**	Tizi-Ouzou	Tigzirt, Tifra	*M. nigricans*.

The slug fauna of western Algeria is less diverse but shows more ancient origins. An ancestral *Drusia* from Europe probably reached North Africa during the Miocene, when a land bridge connected the south of the Iberian Peninsula with North Africa (Manganelli and Giusti 1993), and from there colonised the Canary Islands, giving rise to the endemic genus *Cryptella* P. B. Webb & S. Berthelot, 1833 (Waldén 1984; Hutterer 1990; Hutterer and Groh 1991). The origins of the genus *Letourneuxia* seem similar to those of the genus *Drusia*, and probably has its closest relatives in the genus *Geomalacus* Allman, 1843, which is distributed throughout the Iberian Peninsula (Wiktor and Norris 1991; Patrão et al 2013); moreover, *Letourneuxia* is the only genus of slugs endemic to the Maghreb, which would testify to its long isolation from the arionids of southwestern Europe. The area between Mostagamen and Algiers seems to be a zoogeographical boundary between northeastern and northwestern Algeria. However, it is the least studied region of Algeria for slugs and semi-slugs.

## Conclusions

The present paper provides a synthesis of current knowledge on slugs and semi-slugs from Algeria. Revised distributional data from the literature, combined with the examination of new material, have enabled a more accurate delineation of species ranges. *Ambigolimax parvipenis* is reported here for the first time from Algeria. We propose to reassign the historical taxon *Limax brondelianus* to the genus *Deroceras* as Deroceras cf. brondelianum, based on the examination of the genitalia of topotypes. In addition, two new synonyms are proposed: Amalia cabiliana Pollonera, 1891, syn. nov. as a junior synonym of *Milax gagates* and *Ariunculus pallaryi* Collinge, 1904, syn. nov. as a junior synonym of *Letourneuxia nyctelia*.

This work is intended for specialist taxonomists as well as the broader community of biologists and conservationists interested in assessing local or regional biodiversity and investigating changes in slug fauna composition in relation to land-use dynamics and interactions between native and alien species. However, the study of the Maghrebian slugs remains incomplete, and further research is strongly recommended. *Limax raymondianus* is an uncertain taxon at both specific and generic levels. The status of eight nominal species-level taxa remains unclear: *Deroceras subsaxanum* continues to be regarded as a taxon inquirendum, as topotypical specimens were not available. Likewise, the taxonomic position of *Deroceras* sp., reported by several authors from western Algeria and northern Morocco, remains uncertain (Terver 1839: 9; Morelet 1853: 280; Hesse 1885: 12; Heynemann 1885: 259; Pollonera 1891: 3). The status of *Arion rufus* reported by Aucapitaine (1862) from Thaguemoun’th-ih’addaden (Kabylia, Algeria) also requires clarification, as does the status of five taxa described within the genus *Milax*, i) M. gagates var. mediterranea Cockerell, 1891 (t.l. east Algeria and Sicily); ii) eremiophilus Bourguignat, 1861 (t.l. surroundings of Cherchell); iii) scaptobius Bourguignat, 1862 (t.l. surroundings of Bougie, surroundings of Constantine); iv) insularis var. *algerica*Pollonera (1891) (t.l. Algiers); v) collingei Hesse, 1926 (t.l. Algiers).

## Supplementary Material

XML Treatment for Milax

XML Treatment for Milax ater

XML Treatment for Milax gagates

XML Treatment for Milax nigricans

XML Treatment for Escutiella

XML Treatment for Drusia (Escutiella) deshayesii

XML Treatment for Limacus

XML Treatment for Limacus flavus

XML Treatment for Ambigolimax

XML Treatment for Ambigolimax melitensis

XML Treatment for Ambigolimax waterstoni

XML Treatment for Ambigolimax valentianus

XML Treatment for Ambigolimax parvipenis

XML Treatment for Deroceras

XML Treatment for Deroceras cf. brondelianum

XML Treatment for Deroceras riedelianum

XML Treatment for Vitrina letourneuxi

XML Treatment for Daudebardia

XML Treatment for Daudebardia (Daudebardia) brevipes letourneuxi

XML Treatment for Daudebardia (Daudebardia) rufa atlantica

XML Treatment for Letourneuxia

XML Treatment for Letourneuxia nyctelia

XML Treatment for Testacella

XML Treatment for Testacella (Testacella) fischeriana

XML Treatment for Testacella (Testacella) riedeli

## Additional information

### Use of AI

No use of AI was reported.

### Author ORCIDs

Ramdane Ramdini https://orcid.org/0000-0002-1206-8681

Fabio Liberto https://orcid.org/0000-0001-8533-0911

Ghania Sadouk https://orcid.org/0000-0001-7017-7264

Armando Gregorini https://orcid.org/0000-0003-3667-5686

Mariastella Colomba https://orcid.org/0000-0002-8593-9409

### Data availability

All of the data that support the findings of this study are available in the main text or Supplementary Information.
